# Synchronizing stochastic circadian oscillators in single cells of *Neurospora crassa*

**DOI:** 10.1038/srep35828

**Published:** 2016-10-27

**Authors:** Zhaojie Deng, Sam Arsenault, Cristian Caranica, James Griffith, Taotao Zhu, Ahmad Al-Omari, Heinz-Bernd Schüttler, Jonathan Arnold, Leidong Mao

**Affiliations:** 1 College of Engineering, University of Georgia, Athens, GA 30602, USA; 2 Department of Entomology, University of Georgia, Athens, GA 30602, USA; 3 Department of Statistics, University of Georgia, Athens, GA 30602, USA; 4 Genetics Department, University of Georgia, Athens, GA 30602, USA; 5 College of Agricultural and Environmental Sciences, University of Georgia, Athens, GA 30602, USA; 6 Department of Biomedical Systems and Informatics Engineering, Yarmouk University, Irbid, 21163, Jordan; 7 Department of Physics and Astronomy, University of Georgia, Athens, GA 30602, USA

## Abstract

The synchronization of stochastic coupled oscillators is a central problem in physics and an emerging problem in biology, particularly in the context of circadian rhythms. Most measurements on the biological clock are made at the macroscopic level of millions of cells. Here measurements are made on the oscillators in single cells of the model fungal system, *Neurospora crassa*, with droplet microfluidics and the use of a fluorescent recorder hooked up to a promoter on a *clock controlled gene-2* (*ccg-2*). The oscillators of individual cells are stochastic with a period near 21 hours (h), and using a stochastic clock network ensemble fitted by Markov Chain Monte Carlo implemented on general-purpose graphical processing units (or GPGPUs) we estimated that >94% of the variation in *ccg-2* expression was stochastic (as opposed to experimental error). To overcome this stochasticity at the macroscopic level, cells must synchronize their oscillators. Using a classic measure of similarity in cell trajectories within droplets, the intraclass correlation (ICC), the synchronization surface ICC is measured on >25,000 cells as a function of the number of neighboring cells within a droplet and of time. The synchronization surface provides evidence that cells communicate, and synchronization varies with genotype.

A central problem in physics is understanding the synchronization of stochastic oscillators^1^,^2^,^3^,^4^,^5^, but this problem is largely unstudied in biology^6^, particularly in the context of circadian rhythms. Most measurements on the biological clock are made on millions of cells to understand the mechanism of telling time^7^. A grand challenge is to determine: (1) the behavior of such oscillators on a single cell level; (2) how the clock really functions; (3) whether or not the clock is stochastic in nature; and (4) whether or not clocks of different cells communicate to overcome their stochastic asynchrony.

While single cell measurements have been made on the clocks of cyanobacterial cells^8^ and on synthetic oscillators in *E. coli* by microfluidics^9^, such measurements have been rare on a eukaryotic clock, but when performed, have uncovered new phenomena about the clock^10^,^11^. While stochastic models of the clock exist^12^ at the single cell level, the empirical question of the importance of stochastic variation in the clock remains unanswered. While some initial synchronization studies have been conducted in tissue culture of neuronal cells from the suprachiasmatic nucleus (SCN) constituting the master clock of mammalian cells^13^ and candidate signaling molecules for synchronization have been identified^14^,^15^, the mechanism of synchronization is missing. The number of single-cell trajectories in such studies is typically 100 or less, precluding a test of a synchronization mechanism. Single cell measurements have yet to be made on one of the most fully explored biological clocks in the model fungal system, *Neurospora crassa*, leaving the grand challenge untackled in this model circadian system^7^. Here we introduce a microfluidics platform to address this grand challenge^16^.

Microfluidic techniques provide a flexible way to manipulate single-cells and to perform various single-cell analyses^17^,^18^,^19^,^20^,^21^,^22^,^23^ because they can handle from nanoLiter to femtoLiter amounts of liquid precisely. Droplet microfluidics especially provides an emerging tool for single-cell analysis to isolate single-cells in their individual environment for manipulation^21^,^24^,^25^. The microfluidic platform developed here is based on the general principle of existing droplet microfluidics systems. It features a cell encapsulation device that loads different numbers of cells into droplets and an observation chamber as capillary tube for cell incubation that helps stabilize the cells (in droplets) for long-term (up to 10 days) observation of the circadian rhythm of a large number (>1000) of isolated cells through a fluorescence microscope.

## Results

### Measurements on single cells in a droplet microfluidics system

**Figure 1:**
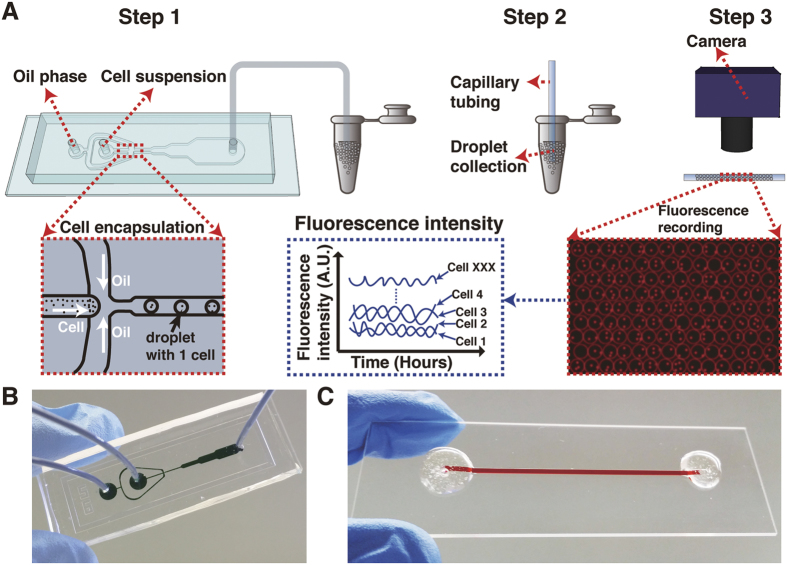
Oscillators of single cells can be measured with a workflow involving droplet microfluidics devices and fluorescent recorders of a clock output gene for over 200 h. (**A**) There are 3 steps for capturing cells in droplets so that fluorescence data can be measured on each cell. In Step 1 cells are encapsulated in droplets by a microfluidics device with flow-focusing geometry. In Step 2: droplets are collected from step 1 into capillary tubing. In Step 3: encapsulated cells are viewed by time-lapse fluorescence imaging, and single cell fluorescence data are extracted. (**B**) Photo of the microfluidics device for cell encapsulation. The channel is dyed green. (**C**) Photo of capillary tubing. The capillary tubing is dyed red.

The workflow of the microfluidic system used here is demonstrated in Fig. 1A. In step 1 a flow-focusing microfluidic device is used to encapsulate cells in droplets. A stream of the *N. crassa* cell suspension meets two streams of fluorinated oil at the intersection as shown in the zoom-in figure entitled ‘Cell encapsulation’. As a result the stream of cell suspension is divided into dispersed droplets with various numbers of cells. Afterwards, the droplets are collected into a capillary tube in step 2. The two ends of the capillary tube are then sealed, and the capillary tube is put onto a motorized microscope stage. A CCD camera is used to record the fluorescence images of the encapsulated cells in step 3. A single layer of droplets is formed in the capillary tube, and the droplets are very stable over ten days (Supplementary video S1), which makes it possible to track the fluorescent intensity of individual cells over time. Figure 1B,C show the photos of the microfluidic device and the sealed capillary tube, respectively. A detailed protocol to record single cell data can be found in a supplementary text.

### Stochastic oscillators

**Figure 2:**
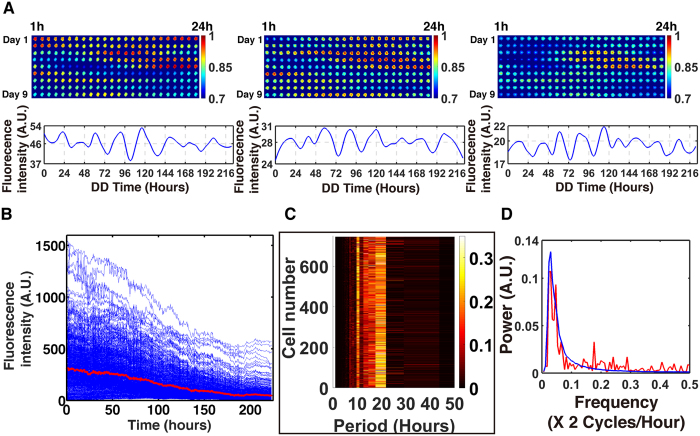
The oscillators in single cells of *N. crassa* are circadian with a period of ~21 h in the dark (D/D), but there is substantial variation in phase and amplitude captured in a stochastic genetic network fitting the single cell clock data. The scales in Fig. 2A (bottom panel of temporal traces) do vary so that it is easier to examine the variation in periods of the three trajectories. Trajectories were normalized and detrended as described in Supplement S2. (**A**) Circadian oscillation of fluorescence data on *ccg-2* gene expression recorder is shown from individual *N. crassa* conidia with substantial variation in amplitude and phase. Scale bar, 20 μm. (**B**) Stochastic variation in 868 cells in one microscope view is shown with only a slight photobleaching effect for the mCherry recorder used. The curve in red is an average of all 868 trajectories (in blue). (**C**) A heat map of 868 cells is shown representing the periodogram of each of 868 cells on the Y-axis and the period, on the X-axis. Yellow is indicative of higher power at a particular period. The period varies about 20–21 h in the heat map. The sum of the periodogram values is used to normalize the power output of each period so that the power can be interpreted as the fraction of oscillators of a particular frequency. (**D**) The average periodogram of a stochastic clock network (in blue) derived from a working ensemble of deterministic models fits the average periodogram of 868 cells (in red) derived from the individual periodograms in panel C.

Here we show the trajectories of 868 single cells each isolated in different droplets in Fig. 2B and measured with a fluorescent recorder (mCherry) driven by the *clock-controlled gene 2* (*ccg-2*) promoter in strain MFNC9^26^. This gene is one of the early identified outputs of the clock in *N. crassa*^27^, and the mCherry fluorescence measures *ccg-2* expression. To remove the complication of synchronization of multiple cells within droplets only isolated cells (singletons) in droplets were initially considered here to measure their stochastic variation in expression. All cells were transferred to the dark (for ten days) to allow circadian rhythms to develop interrupted only briefly during imaging of cells (every 30 min). It is evident that there is substantial variation in the trajectories of *ccg-2* expression in different isolated cells in Fig. 2B. In Fig. 2A there are some sample trajectories. While each sample trajectory in Fig. 2A has a period near 21 h, the phase and amplitude vary. A summary of the periods of all trajectories is captured in the periodograms of each cell in a heat map (Fig. 2C). The principal period is 21 h with limited variation about this mean as expected^26^.

### Measurements of expression on single cells over 10 days

One of the advantages of the microfluidics device is the ability to measure expression on each of 868 single cells over 10 days in Fig. 2. Their fluorescence varied rhythmically over time. Droplet stability was dependent on the surfactant used to coat the droplets^25^, and the stability of cell location was in part due to the microfluidics device as well as media derived previously^28^. A recently developed automated cell counting technique was used to verify that 80% +/− 2% of the cells were still viable at the end of the ten day experiment (Supplement S1)^29^.

### Characterization of the stochastic oscillator in each cell

In each trajectory there are two components of variation in fluorescence, stochastic variation in *ccg-2* gene expression and variation due to experimental detection noise; the stochastic variation can be further decomposed into intrinsic variation in *ccg-2* expression and extrinsic variation due to other cellular components^30^. Both sources of variation, stochastic and that due to experimental detection noise, can be quantified (see Supplement S2), with a control experiment in which cells are replaced with fluorescent beads of diameter, comparable to the mean size of macroconidial cells. The latter was measured with automated cell counting of 636 cells to be 8 μm (Supplement S1). The stochastic trajectories do display a slight negative trend due to the photobleaching of the mCherry recorder gene. To control the photobleaching, the time between measurements was limited to 30 minutes. The result was 480,000 fluorescent measurements of *ccg-2* expression on the circadian oscillators of ~1,000 cells.

It would be desirable to know the sources of variation in the rhythms of individual cells. First, a number of steps were taken to reduce the variation due to experimental error in trajectories. For example, the depth of the observation chamber in Fig. 1 was 50 μm in size to prevent cells from drifting in or out of focus. Rhodamine B was introduced as an internal standard so that the measured fluorescence of cells was made relative to the Rhodamine B standard to reduce experimental error. The media was selected to inhibit cell division, and direct observation was used to confirm no cell division occurred during the 10 day experiment. A workflow including a quality control filter was applied to ensure that particle (cell) tracking from frame to frame was carried out correctly (supplementary Text S1). Using an automated cellometer (Supplement S1), we observed that the mean cell size was 8 μm, and so a 10 day control experiment with 9.94 μm fluorescent beads replacing cells was conducted in the same way, allowing us to estimate separately the variation due to experimental error from the stochastic variation between genetically identical cells.

To know the sources of variation in Fig. 2B and its mechanism we setup a stochastic chemical rate equation model derived from the ensemble of deterministic working models described previously^31^. The initial guess (from the working deterministic ensemble) for the rate constants and initial conditions of the stochastic clock network were then used to initialize a Metropolis Monte Carlo (MC) method (Supplement S2) to search for an ensemble of ~1,000 stochastic models with an average periodogram fitting the observed average periodogram in Fig. 2D. At each step in this ensemble method it was necessary to propose an update to the model and to simulate quickly 1,024 single cell trajectories (as in the real experiments) using the Gillespie Algorithm^32^ (1024 simulated trajectories of 10 days in 2 seconds), implemented on a general-purpose graphics processing unit (GPGPU) (see Supplement S2). Over 165,000 such proposals were accepted or rejected according to the similarity of the fitted periodogram (computed from the 1,024 simulated cell trajectories of the proposed model) to the observed one in Fig. 2D (see Supplement S2), and in this way the fitted periodogram was updated or stayed the same. The resulting model ensemble fitted quite well (Fig. 2D) to the observed average periodogram of 868 singleton cells, and five frequencies (including one subharmonic) explained over 53% of the variation in the observed periodogram in Table 1. The five frequencies around 21 h are highly significant by an F-test relative to the stochastic + experimental error variation.

Westermark *et al*.^33^ recognized the importance of an error model for single cell measurements. The stochastic model was then coupled to an experimental detection error model for single cell measurements with substantial empirical basis (see Supplement S2)^34^. While the stochastic model served as a source for the stochastic variation, the error model served as a source for the experimental error from detection noise in the fluorescent cell signal. The error model was identified with six bead experiments. Both sources of noise were propagated to the periodogram (see Materials and Methods), where the stochastic variation and variation due to experimental error could be partitioned (see Supplement S2). A sizeable > 94% of the variation was stochastic variation (as opposed to experimental detection noise) in expression between cells.

Having separated the stochastic variation measured in cells over ten days (Fig. 2B) from the experimental error measured in control bead experiments (using fluorescent 9.94 μm beads), we wished to know if the oscillators were circadian. A separate periodogram was calculated for each cell in Fig. 2C (see Materials and Methods), and the average periodogram has a peak at 21 h in Fig. 2D as expected^26^ from both race tubes and fluorometry at the macroscopic level. The oscillators of individual cells were circadian, but they are clearly not in phase from Fig. 2B. As an example of this, the periodicity of the average trajectory (Fig. 2B in red) is very weak, if visible at all, but the periodograms of individual cells display a strong peak in the periodogram near 21 h in Fig. 2C. As with the suprachiasmatic nuclei (SCN) of mammalian cells, *N. crassa* cells have cell-autonomous oscillators^35^. The character of this cell autonomous oscillators in *N. crassa* is different from those in the SCN^36^. Most isolated cells in the SCN display no oscillations unless they express the paralog, PER2, to FRQ^37^ (Fig. 1); however, most isolated cells in *N. crassa* are oscillatory (Fig. 2C).

With a model in hand fitting the single cell data quite well (χ^2^ = 130.231, df = 137, P = 0.65), it was then possible to simulate trajectories of the fitted model to determine the source of stochastic variation. It was found to arise in part from the transcriptional bursting of the core clock genes themselves^38^. The core clock genes, *white-collar-1* (*wc-1*) and *frequency* (*frq*) with *ccg-2* gene coming on and off randomly generated the variation in phase of cell trajectories (supplementary text, Fig. S1). The counts of mRNAs from *ccg-2* from the fitted clock model are also quite low with their concomitant stochastic variation; the average mRNA level of *ccg-2* mRNA is 129 molecules/cell +/− 27 molecules/cell averaged over 240 h and 1,024 detrended Gillespie trajectories (see Supplement S2). The average protein level of CCG-2 is only 264 molecules/cell +/−68 molecules/cell averaged over 240 h and 1,024 detrended Gillespie trajectories. Our hypothesis then for explaining the stochastic variation is that the random activation of core clock genes in a single cell leads to the stochastic variation in phase seen in Fig. 2B.

### The stochastic model for the clock is validated by an independent test using the phase of the circadian single cell oscillators

**Figure 3:**
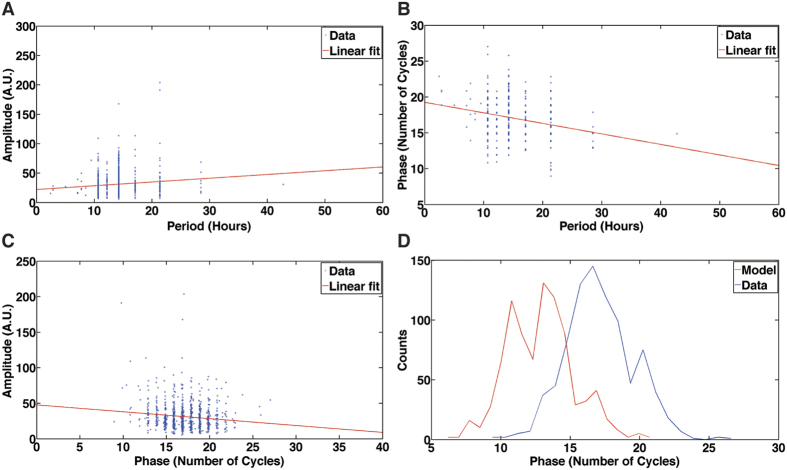
The stochastic clock model for single cells is validated by an independent test of the model involving predicting the observed phase of 868 single cell oscillators from the model. (**A**) Plot of amplitude vs. period for 868 single cell oscillators in Fig. 2B. The amplitude is the square root of the maximum power in the periodogram. The period is 1/2*π*f_l_, *i.e.,* the inverse of the frequency f_l_ at which the periodogram is maximum in power. The correlation (r) of amplitude and period is r = 0.1095 (Fishers z = 0.1099, P < 0.001)^79^. The Spearman rank correlation (r_s_) is r_s_ = 0.1101 (P < 0.01)^40^. The straight line regression of amplitude on period (in red) is also shown. (**B**) Plot of phase vs. period for 868 single cell oscillators in Fig. 2B. The discrete Hilbert phase 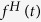 at time t is calculated from the Hilbert transform of each cell’s trajectory after subtracting its mean over time^44^. The phase of an oscillator plotted here is defined as 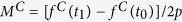 in units of cycles, where 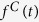 is the continuous Hilbert phase at time t. The phase can be thought of as the number of cycles, which a single cell oscillator completes in the time interval t_1_–t_0_ = 230–60 in units of half hours. The continuous Hilbert Phase 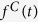 is defined recursively by 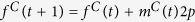 where 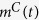 is the argument m that minimizes 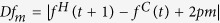. The correlation (r) of phase and period is r = −0.1986 (Fishers z = −0.2013, P < 0.001)^79^. The Spearman rank correlation (r_s_) is r_s_ = −0.1376 (P < 0.001). The straight line regression of phase on period (in red) is also shown. (**C**) Plot of Amplitude vs. phase for 868 single cell oscillators in Fig. 2B. The correlation (r) of amplitude and phase is r = −0.1226 (Fisher’s z = −0.1232, P < 0.001). The Spearman rank correlation (r_s_) is r_s_ = −0.0880 (P < 0.02). The straight line regression of amplitude on phase is shown (in red). (**D**) Histogram of the phase of 868 single cell oscillators in Fig. 2B and 868 Gillespie trajectories of the stochastic model. The Gillespie trajectories from the model were computed as described in Supplement S2. The phase is functionally independent of the periodogram. Trajectories in both model and data are detrended (See Supplement S2).

Some have hypothesized that the period of each circadian single cell oscillator should be independent of its amplitude^39^. We hypothesize that the amplitude and period of each single cell oscillator should be positively correlated because they are simply related features of the periodogram of each single cell oscillator (Fig. 2C). We also predict that there should be an inverse relation between phase (*i.e*., the number of cycles completed in a fixed time period by a single cell oscillator) and the period of the oscillator. Since phase and period are expected to have a negative relation and period is expected to have a positive relation to amplitude, we also predict that phase should have a negative relation to amplitude. Plots of the phase, amplitude, and period of each single cell oscillator support our hypotheses (Fig. 3). Period and amplitude are significantly positively correlated as expected (Fig. 3A), and amplitude and phase are significantly negatively correlated as expected (Fig. 3C). Our ability to detect a correlation between period and amplitude (Fig. 3A) is probably due to observing 3.4X as many single cell oscillators as the earlier experiment^39^.

Each cell trajectory has a phase and periodogram in Fig. 2C,D. When the periodogram is calculated for a cell trajectory from Eq. (S51) (Supplement S2), the phase is lost when the modulus is taken. In that the phase is functionally independent of the periodograms of single cells used to fit the stochastic model (Fig. 2D) and hence not used in the stochastic model fitting, this provides an opportunity for an independent test of the stochastic clock model. A histogram of phase was constructed from the observed trajectories of 868 single cell oscillators and compared with the phase constructed from 868 Gillespie trajectories under the fitted model (Fig. 3D). There is good agreement between these two histograms, validating the stochastic model of the clock for single cells (Fig. 3D). The only difference is that the mean (+/−two standard errors) of the oscillator phases observed (17 +/− 0.16 cycles/85 hours) is slightly higher than that predicted by the stochastic model (13 +/− 0.16 cycles/85 hours). A Kolmogorov-Smirnov 2-sample test^40^ comparing the two phase differences after subtracting means barely reaches significance (P = 0.02).

### Synchronization measures of the circadian stochastic oscillators

In the face of substantial variation in the circadian stochastic oscillators within single cells (Fig. 3), the question remains: how do they synchronize to produce the regular biological clock observed at the macroscopic level by race tubes or fluorescent or luciferase recorders? At some point the ensemble of oscillators overcomes stochastic asynchrony. In order to demonstrate this synchronization we needed to be able to measure their collective behavior^41^. For example, Garcia-Ojalvo *et al*.^42^ have suggested one way of measuring synchronization, namely in our context, the variance in the mean fluorescence of cells within a droplet divided by variance in fluorescence of a single cell (*i.e*., the variance of the red curve over time divided by the variance in individual trajectories in blue in Fig. 2B over time). They analyzed this measure in the context of a system of coupled repressilators related to our own working clock network^43^. What was missing from this measure is accounting for the between droplet variability in our experiment.

For other systems an array of synchronization measures have been utilized, such as the maximum of the cross-correlation between cells (with respect to frequency), mutual information, phase synchronization extracted by either Hilbert or Wavelet transform, or an index based on a circular variance^41^,^44^. A careful study of the utility of such measures has led some authors to conclude there is no universally best measure of synchronization, but the measure needs to be tailored to the problem at hand^44^. The focus of most of these measures is on phase synchronization.

A good synchronization measure should not only capture similarity in the phase of circadian oscillators, but also the similarity in their amplitude and period. Second, the measure should measure the synchrony or similarity of cell trajectories within a droplet relative to the variability between droplets. Third, the measure should be easily interpretable, as is the measure suggested previously^42^. Fourth, the measure should have well defined statistical properties to allow inference about the systems of coupled oscillators.

One such measure with all of these properties is the *intraclass correlation*^45^,^46^. Consider one time point in the microfluidics experiment. Let X_ij_ be the fluorescence of the jth cell in the ith droplet at one time point when there are *a* cells per droplet. We assume that the measurements X_ij_ have the following covariance structure^45^:


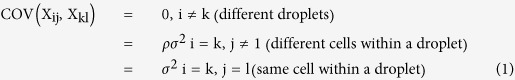


The covariance (COV) is defined over all cells j in droplet i of size *a* at a particular time point.

In this variance components model^45^ the intraclass correlation is ρ, the correlation between different measurements within a droplet. This measure has been used for over 150 years^45^, particularly in twin studies as a measure of heritability^47^. The variance *σ*^2^ in one cell within one droplet at one time can be estimated by observing cells in replicate droplets. The partitioning of fluorescence variation is summarized in an analysis of variance (ANOVA) in Table 2 45. The total variation in fluorescence SS_T_ was partitioned into a between droplet sum of squares (SS_B_) and a within droplet sum of squares (SS_W_) in Table 2 such that SS_T_ = SS_B_ + SS_W_. By setting the estimated mean squares (EMS_B_ and EMS_W_) equal to their expectations in Table 2, we obtain two equations in two unknowns (*σ*^2^and *ρ*) in Table 2 to arrive at a sample estimator of the intraclass correlation (ICC):


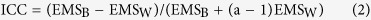


The measure ICC in Eq. 2 estimating ρ captures similarity of measurements within droplets relative to the variation between droplets. This measure of synchronization has two simple interpretations: (1) the correlation between cell measurements within a droplet from Eq. 1; (2) the fraction of variation explained within droplets relative to between droplets from Table 2.

### A deterministic quorum sensing model for the circadian oscillators

**Figure 4:**
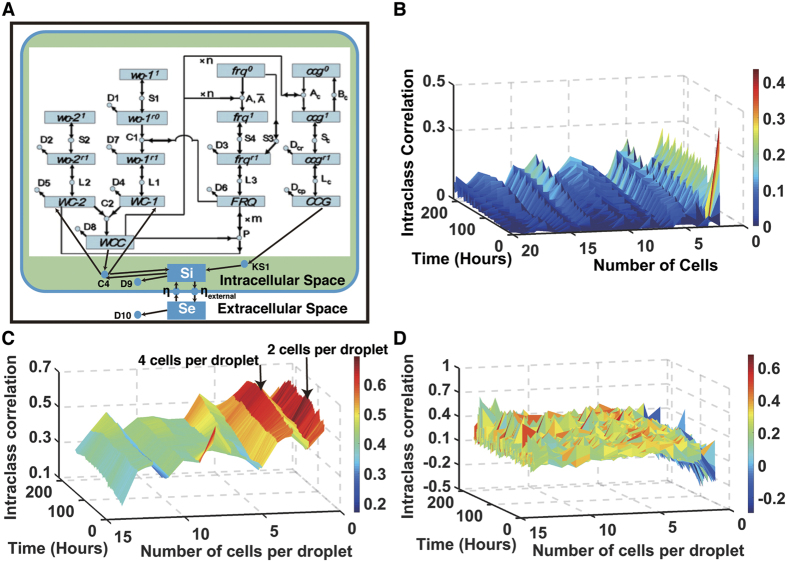
Conidial cells communicate the state of their oscillators to each other within a droplet. (**A**) A simple deterministic quorum sensing model is developed to interpret the similarity of trajectories within a droplet relative to the variation between droplets. Within each cell the oscillator network is hypothesized to be that of the working ensemble fitting the data at the macroscopic level of 10^7^ cells. Boxes denote reactants and products in the network; circles denote reactions. Arrows pointing into reactions denote reactants, while arrows pointing out indicate products. Lines with arrows on both ends connect catalysts to a reaction. Reaction and molecular species labels in the network denote parameters in the model, rate coefficients and initial conditions, respectively. A new feature of the model is a *clock-controlled gene* (*ccg*) that makes a signaling molecule S_i_ within the cell, which then diffuses in or out at a rate *η* or η_ext_. The signaling molecule S_i_ interacts with WC − 1 and WC − 2 to slow the production of WCC to synchronize the clocks of different cells. Modified from earlier network diagram^43^. (**B**) The synchronization surface is shown for this simple quorum sensing model with new parameters given in Eq. (3) and remaining parameters published previously^77^ and released in sourceforge.net under the keyword, vtens_EI_clock1 and in Table S1. Synchronization or more generally similarity of cell trajectories within droplets is measured by the intraclass correlation (ICC) relative to the variation of cell trajectories between droplets. The ICC is shown as a function of time and number of cells per droplet for the model. (**C**) The ICC synchronization surface of the data on 7,903 cells is quite similar in structure to that of the quorum sensing model. (**D**) As a control neighboring cells within droplets are replaced at random with strangers that have experienced no neighbors. The resulting surface has no structure, providing prima facie evidence of cell-cell communication within droplets.

Kreuz *et al*.^44^ have provided strong evidence that the utility of a synchronization measure depends strongly on the context for its use. Thus, we developed a relatively simple quorum sensing model of communication between cells within droplets. This model has some of the features used previously^42^ and has been referred to as a mean-field model for coupled oscillators^48^. Other forms of cell to cell communication may be relevant that involve contact between cells or distance between cells^3^,^49^,^50^. The model is summarized in Fig. 4A. Only a few parts in Eq. 3 (in red) are new additions to a previously working model ensemble for the clock^31^:


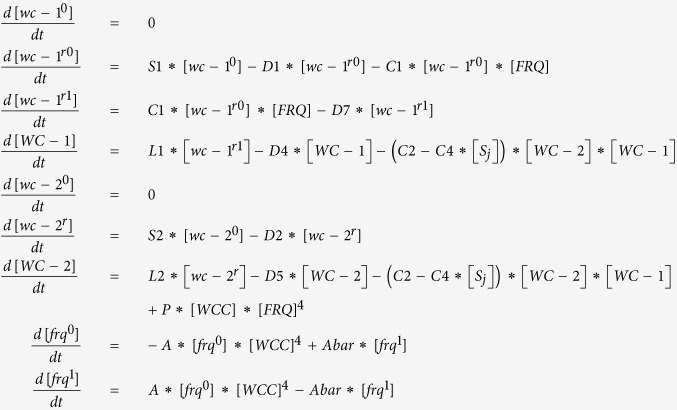



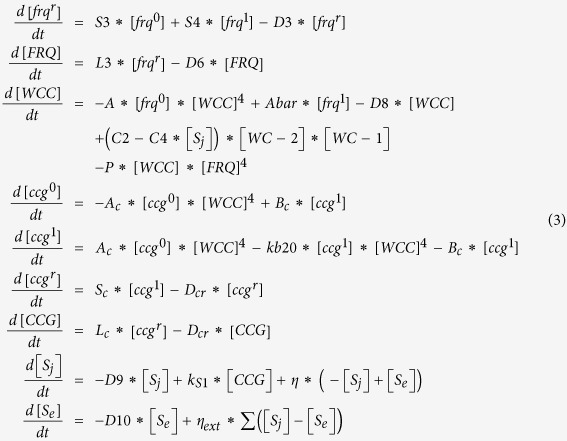


where D2 = 0.0001, *η* = 100, *η*_*ext*_ = 1.44, D9 = 26, D10 = 4, C4 = 0.8, k_S1_ = 5 × 10^9^. A source for the remaining coefficients is given in the legend of Fig. 4 and in Supplement S2. There is an implicit index j = 1, …, a on all species in Eq. 3, and the sum in the last equation is over all cells in a single droplet. Also the parenthetical terms including C2 and C4 are implicitly multiplied by a Heaviside step function to keep them non-negative.

This quorum sensing model (Fig. 4A) is based upon a mean field assumption of instantaneous and uniform diffusion of the signaling molecule (sometimes referred to as the autoinducer) within a droplet. In our model, this signal is formed by a “*clock-controlled gene (ccg)*” and is, consequently, an oscillating output driven by the central clock network specified by Eq. 3. This signal moves into and out of the cell at rates (*η* and ***η***_*ext*_) dependent upon the relationship between internal and external signal concentration ([S_j_] and [S^e^], respectively) as well as based upon the difference in volume of the cell compared to its droplet microenvironment (incorporated into the η_**ext**_ parameter). The signal in the media decays at a rate D10.

There are a number of possible models for how the signal interacts with the oscillator in each cell^51^. When the model in Eq. 3 is linearized, it then becomes similar to the repressilator^43^. This fact suggested that the coupling of cells might be taken to be similar to that of coupled repressilators^42^. A remaining question is what clock gene interacts with the incoming signal. The WCC complex interacts with Flavin Adenine Dinucleotide or FAD to receive the light signal and has a number of additional domains that may interact with other incoming signals^52^. These facts suggested WCC as the interactor with the hypothesized quorum sensing signal in Eq. 3. The sign of the interaction was determined by the relative ease of synchronizing oscillators in different cells by using the signal to inhibit WCC production. The signal within a cell interacts with [WC − 1] and [WC − 2] in Eq. 3 in order to slow the formation of [WCC] which slows the production of [CCG] and [S_j_], thereby closing a coupling negative feedback loop.

Two classes of negative feedback models have been used for circadian rhythms, protein sequestration models and Hill Type transcriptional repression models^53^. In protein sequestration models it is assumed that that the repressor (FRQ homolog) binds stochiometrically in a 1:1 ratio with the transcriptional activator(s) (WCC homolog). In Hill Type transcriptional repression models the repressor (FRQ homolog) binds upstream of the activator as a multimer. These Hill Type models have been argued to be similar in structure to models with phosphorylation dependent repression of the activator^53^. Phosphorylation-dependent repression of the activator cannot currently be distinguished experimentally from the mechanism of FRQ inactivation of WCC in Eq. 3^43^. The protein sequestration models are predicted to apply to Drosophila and mammals; the Hill Type models are predicted to apply to cyanobacteria and *N. crassa*^51^,^53^.

Each class of negative feedback models with cell-to-cell communication makes fundamentally different predictions about how the period of the single cell oscillators behave as a function of the number of communicating oscillators^53^. In protein sequestration models the mean period across single cell oscillators is predicted not to change with the number of communicating oscillators, but the variance in period across single cell oscillators should decrease with the number of communicating oscillators. In contrast with a Hill Type model the mean period across single cell oscillators is predicted to change with the number of oscillators. In mammals the prediction of stable mean period and decreasing variance in period with increasing number of communicating single cell oscillators has been experimentally confirmed^11^,^14^. The prediction for *N. crassa* was confirmed here as well in a ten day microfluidics experiment with the reference strain MFNC9 – the mean period of the single cell oscillators shifted significantly as the number of oscillators per droplet was increased (Table 3). The variance in period also declined significantly with the number of cells per droplet (Table 3), as in mammalian systems. This decline in variance in period across single cell oscillators within droplets could be understood by the examination of synchronization of these oscillators within droplets.

### Synchronization of stochastic circadian oscillators

Under the null hypothesis of mean field quorum sensing we established an expectation for the synchronization surface (Fig. 4B) based on a working ensemble of deterministic models for the clock. We then constructed the synchronization surface for the ten day microfluidic experiment involving 7,903 cells as a function of time and number of cells per droplet (Fig. 4C).

As can be seen in Fig. 4C, the synchronization surface was quite similar to that of the mean field quorum sensing model. Both surfaces (Fig. 4B,C) increase along the time axis for an even number of cells per droplet.

**Figure 5:**
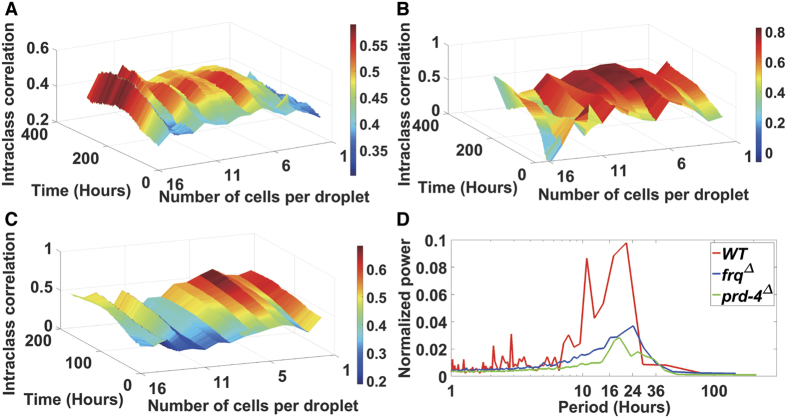
The synchronization surface (ICC) is a function of genotype. (**A**) A replicate of the ICC surface with >25,000 cells was constructed^46^ and resembles Fig. 4B. (**B**) The deletion of *prd-4* in *prd-4* Δ*,ccg-2p:mCherry* results in increased synchronization. (**C**) The deletion of the oscillator *frq* Δ*,ccg-2p:mCherry* leads to a more even synchronization surface. (**D**) The power at 21 h in the average periodogram over singletons for the *frq*^*Δ*^ is reduced ~3-fold relative to MFNC9, but is not eliminated, suggesting a FRQ-less oscillator. Period (Hours) is plotted on a log scale.

It is natural to ask whether or not this synchronization surface is real and significant. First, as a confirmation we replicated the experiment leading to the synchronization surface (Fig. 4C) with over 25,000 cells, producing a surface of similar structure from >12 million time points (Fig. 5A). As another control for each droplet with more than one cell, the fluorescence value was replaced randomly with replacement by the fluorescence value of a singleton at the same time point. At each time point the minimum number of singletons in the pool was 193. This sampling with replacement was done for all droplets with multiple cells at all time points. In other words, neighbors were replaced with strangers in each droplet. The result was the synchronization surface in Fig. 4D. There was no structure to this surface, unlike Fig. 4B,C. This synchronization surface was replicated 20 times, replacing neighbors with strangers, with the same result. This is prima facie evidence that the cells within droplets are communicating.

There were several other interesting features to the synchronization surface Fig. 4C. For certain droplets with a specified number of cells, there was an upward trend in time in the intraclass correlation, as might be expected as synchronization evolves. We examined how this synchronization surface may be originating. Using sampling with replacement of neighbors by strangers again, we found that the more traditional measures of phase synchronization behaved as expected between the experiment (Fig. 4C) and control (Fig. 4D). Along the ridges of Fig. 4C of 2 or 4 cells per droplet, the synchronization measure of Garcia-Ojalvo *et al*.^42^ or of Kreuz^44^, was high, Fig. S2, but hovered around 0.5 or 0, respectively for the control with strangers replacing neighbors (supplementary Text). The Kuramoto order parameter K (*i.e*., another synchronization measure) also displayed the ridges and coherence of the single cell oscillators (Fig. S3), but was less informative than the ICC surface^54^. This order parameter K involves time averages over each droplet with a particular number of cells (a) that eliminate the structure visible in the time dimension of the ICC surface. There was also a ridge and valley structure to the synchronization surface (ICC) both for the model and data. It is clear that we can measure synchronization with the microfluidics device and that the behavior of cells varies not only with time but neighborhood size as well.

As a final control on the choice of synchronization measure ICC in Eq. 2, we let the dynamics of each cell’s fluorescence be governed by the classic Kuramoto phase-locking model with the data structure of the primary ten day experiment^1^. The resulting synchronization surface Fig. S4 (see Supplement) looked very similar to the data and quorum sensing model in Fig. 4B with a little delay to synchronization due to a local phase-locking assumption in the Kuramoto model.

### The synchronization surface varies with genotype

**Figure 6:**
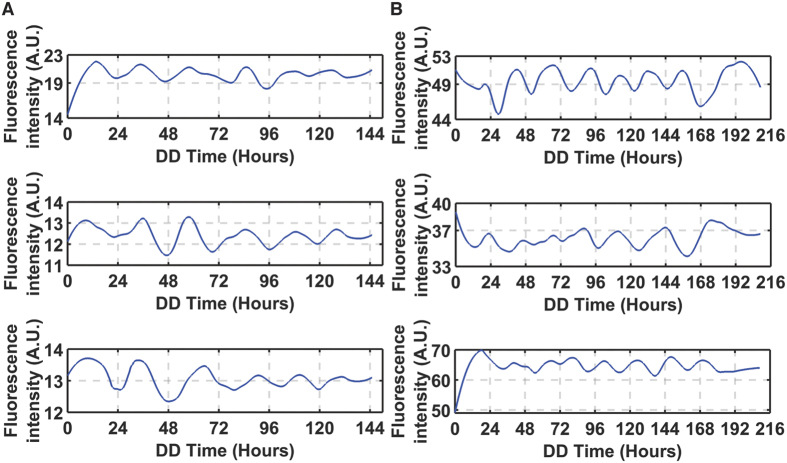
Randomly picked trajectories of (**A**) *frq* Δ*,ccg-2p:mCherry* and (**B**) *prd-4* Δ*,ccg-2p:mCherry* over 144 and 216 h, respectively. These trajectories are normalized and detrended.

The synchronization surface (ICC) is a reaction norm, which is not only a function of the “social environment” of a cell through neighborhood size, but also of genotype. In a genetic screen we have identified a gain-of-function mutation that increases synchronization. The gain of function mutation is from the knockout, *prd-4* Δ*,ccg-2p:mCherry*. As previously reported^55^, this mutant reduces the period by ~3 h to 19 h (Fig. 5D). Some single cell trajectories are also given (Fig. 6). This gene probably acts as a checkpoint kinase in mitosis^55^ (and hence affects conidiation over time, the main clock phenotype assayed in race tubes). Our initial hypothesis is that *prd-4* acts to monitor for DNA damage, a quality control operation that likely introduces a delay between cells. The result is some asynchronization. In *prd-4* Δ*,ccg-2p:mCherry* there is no such delay introduced, and much higher synchronization is achieved at the cost of no proof-reading of the genome (Fig. 5A vs. Fig. 5B).

In contrast *frq* Δ*,ccg-2p:mCherry* knockout has a period of 24 h, and the synchronization surface is responding to a loss of clock function. The *frq* Δ*,ccg-2p:mCherry* was confirmed not to band in race tubes. Synchronization surface looks more even than the MFNC9 genotype (Fig. 5C vs 5A or 5B). Consistent with experiments done on *Synnecocchus elongatus*, knockout of a clock gene does not completely remove oscillations, only reduces their power by ~3-fold (Fig. 5D)^22^. Some examples of *frq* Δ*,ccg-2p:mCherry* trajectories with low amplitudes are given (Fig. 6). This outcome is consistent with earlier data suggesting a second weaker oscillator in *N. crassa* other than the one that is *FRQ* based. In this case the residual synchronization may be due to another FRQ-less oscillator^56^. Alternatively, the FRQ-based oscillator may be orchestrating cell-to-cell synchronization in a light response and may be secondary to the FRQ-less oscillator^57^. This alternative hypothesis cannot be ruled out at this stage as well.

**Figure 7:**
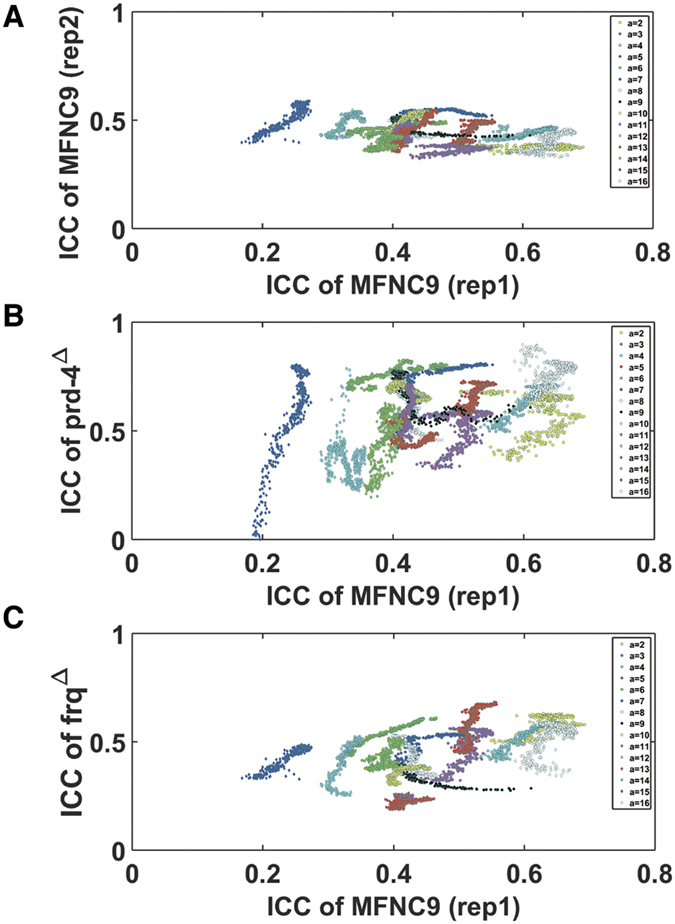
The synchronization surface for the *prd-4* knockout is significantly different from that of the reference strain, *ccg-2p:mCherry* (MFNC9). (**A**) Plot of replicate 2 of *ccg-2p:mCherry* (MFNC9)’s synchronization surface (ICC) in Fig. 5A against replicate 1 of *ccg-2p:mCherry* (MFNC9)’s synchronization surface (ICC) in Fig. 4C. This plot is the negative control. (**B**) Plot of *prd-4* Δ*,ccg-2p:mCherry*’s synchronization surface (ICC) in Fig. 5B against replicate 1 of *ccg-2p:mCherry* (MFNC9)’s synchronization surface (ICC) in Fig. 4C. (**C**) Plot of *frq* Δ*, ccg-2p:mCherry*’s synchronization surface (ICC) in Fig. 5C against replicate 1 of *ccg-2p:mCherry* (MFNC9)’s ICC synchronization surface in Fig. 4C.

The significance of genotypic differences in synchronization (ICC) can be assessed by plotting one genotype’s synchronization surface against another genotype’s surface (Fig. 7). The plots in Fig. 7 are based on a total of ~50 million time points. The synchronization values are organized into stripes of different numbers of cells per droplet (*i.e*., *a*). For example, the ICC surface of *prd-4* Δ*,ccg-2p:mCherry* is seen to reach much higher ICC values than the reference strain MFNC9 (Fig. 7B vs. Fig. 7A). Significance can be assessed by regressing the ICC surface on another ICC surface. Regressing replicate 2 (Fig. 5A) on replicate 1 (Fig. 4C) allows us to capture the stochastic variability in the measurements with Fig. 7A acting as a negative control for comparisons of other genotypic ICC surface pairs. These regressions (Fig. 7) capture at least 60% of the variation in a particular surface, depending on the surfaces compared (Table 4). From the regression of surface y = ICC_2_ on x = ICC_1_, the mean value of synchronization measure (ICC_2_) with standard errors can be computed as a function of the number of cells per droplet (*a*) (Table 4). The 95% confidence intervals about the mean synchronization (ICC) for a given number of cells per droplet (a) in Table 4 are non-overlapping in comparing *prd-4* Δ*,ccg-2p:mCherry* (Fig. 7B) and replicate 2 of MFNC9 (Fig. 7A) and hence highly significant. From the comparison of these plots we also see that different genes lead to different levels of variation in synchronization relative to the reference surface (Fig. 4C). For example, not only does *prd-4* Δ*,ccg-2p:mCherry* reach higher levels of synchronization (Fig. 7B) there is also more variation in synchronization than that in the reference strain MFNC9 (Fig. 7A).

## Discussion

The experimental study of stochastic coupled oscillators in biology is largely missing, particularly in the experimental study of circadian rhythms^6^. There are at least three theories on how cells in a circadian system might synchronize.

One non-intuitive theory is that single cell oscillators may experience stochastic resonance, leading to their synchronization^58^,^59^,^60^. Stochastic behavior of cells in tissue culture may actually lead to increased synchronization^35^. The stochasticity in expression of single cells of *N. crassa* has been quantified here and is substantial (>94% of the variation in single cells). Here we have shown that synchronization does depend on genotype (Fig. 5). Under this stochastic resonance hypothesis an explanation would be needed for why some genotypes improve synchronization through varying the noise in the single cell oscillators. Here the genotype of *prd-4* Δ is likely to act to improve synchronization by decreasing the phase noise in the oscillators by removal of a cell cycle checkpoint^55^. Another non-intuitive possibility is that the removal of the checkpoint actually increases phase noise to improve synchronization. A key feature of the stochastic resonance hypothesis is that synchronization varies non-monotonically with stochastic intracellular noise and that there is a local maximum in synchronization as the stochastic noise is varied. In Fig. 5D the periodogram signal strength near ~24 h is ***weaker*** for *prd-4*^Δ^ than for either WT or *frq* Δ (red = WT, blue = *frq* Δ, green = *prd-4* Δ), but the synchronization is higher. Let us suppose the reference strain (MFNC9) sits to the left of a synchronization maximum as a function of intracellular noise. If the reference strain (MFNC9) were then shifted to a higher intracellular noise level by the *prd-4* deletion, then we would see more synchronization, as seen (Fig. 5B). If we consider instead the synchronization as a function of the phase noise as measured through the phase standard deviation (SD = 4.41 cycles) of the *prd-4* deletion versus that of reference strain MFNC(SD = 2.40 cycles), increased phase noise would move us towards the synchronization maximum as well. The results in Fig. 5 are then consistent with the stochastic resonance hypothesis. There may be other genes that also act to improve synchronization through increasing the intracellular noise of single cell oscillators, such as *frq* Δ. The microfluidics platform enables the quantification of the variation introduced into each single cell oscillator by a particular genotype and its effect on synchronization (Fig. 5).

A second theory is that discrete replication events drive the coupling of circadian system and the cell cycle^61^. The circadian system could become phase-locked to the cell cycle. The evidence against this theory of synchronization is that the cells in this study do not experience any cell division (Supplementary Video S1).

The final theory of synchronization favored here is that cells communicate the state of their clocks through some communication mechanism, such as quorum sensing^48^ (Fig. 4A). We have provided strong evidence that conidial oscillators communicate the state of their oscillators within droplets (Figs 4C and 5A). A prediction of this model is that as the number of cells per droplet (*a*) increases, one should observe a rise in synchronization^42^. A trend of this sort is seen in Table 4. Another prediction of this theory is the structure of the synchronization surface (ICC) (Fig. 4B), which resembles that measured (Fig. 4C). The alternating structure to synchronization with an even number of cells per droplet versus an odd number of cells per droplet is a new phenomenon. We eliminated the cause being the synchronization measure (Supplement S2 in Fig. S2). We also found this alternating structure in different models describing the data (Fig. 5 and Fig. S3). We anticipate further detailed analysis of the ICC surface in these models will provide an explanation for the phenomenon.

None of these mechanisms of synchronization are mutually exclusive. For example, Ulner *et al*.^62^ combined the stochastic resonance hypothesis with quorum sensing in a model to explain cell synchronization in the suprachiasmatic nuclei under light of randomly varying intensity in mammals. Further experiments will uncover the exact mechanism of communication through manipulation of cells in their droplet environment.

There are a number of extensions needed. *N. crassa* has an interesting life cycle^63^. We have focused on single cells or conidia to take maximum advantage of high-throughput cell isolation by microfluidics in Fig. 1 to measure fluorescence on 25,000 cells simultaneously over ten days (Fig. 5A). We selected a media so that cells did not germinate, divide, or fuse (See Video S1). It is in this stage that we were able for the first time to demonstrate circadian rhythms in expression of *ccg-2* at the single cell level. These conidia can be multi-nucleate^63^, and we have not examined intracellular communication, only intercellular communication. These conidia ultimately germinate and produce filaments as a subsequent life stage. Microfluidics devices also provide a tool for examination of these filaments for circadian rhythms^64^,^65^. It will be very interesting to see how the stochasticity in circadian rhythms changes at other life stages in an examination of period, phase, and amplitude variation change under intracellular communication (Fig. 3). Even more interesting would be to examine how the synchronization surface changes as the cells divide during filamentous growth and experience both intracellular and intercellular communication. To reconstruct the synchronization surface will require on the order of 50,000,000 time points (Fig. 7) to make such comparisons, which necessitates both high-throughput and high-resolution microfluidic measurements^64^.

Palma-Guerrero *et al*.^49^ recently described a population genetic approach through a genome-wide association study (GWAS) for isolating genes involved in cell-to-cell communication in *N. crassa*. Their GWAS produced one neuronal calcium sensor homolog (*cse-1*) and six other candidate genes. The experiments here provide a much more direct way to assay communication at the single cell level in which the environment of a droplet can be manipulated in a variety of ways. This combination of a top-down and bottom-up approach should provide a means to uncover new communication pathways between cells in fungi.

What is missing from single cell measurements on biological processes, such as circadian rhythms, is a universal system of measurement. Such a system should be able to identify both cellular stochastic variation and detection noise (*i.e*., experimental error). The network model needs to be stochastic to generate the intracellular stochastic variation^30^, and the detection model needs to be a well-grounded physical model to generate detection noise. Second, the cellular measurements should ideally be normalized against an internal control to capture uncontrolled factors. Such a system should also be able to propagate the sources of error (stochastic intracellular noise and detection noise) to the statistics used to test biological hypotheses of interest. Finally, the sources of error should have quantifiable effects on these statistics.

**Figure 8:**
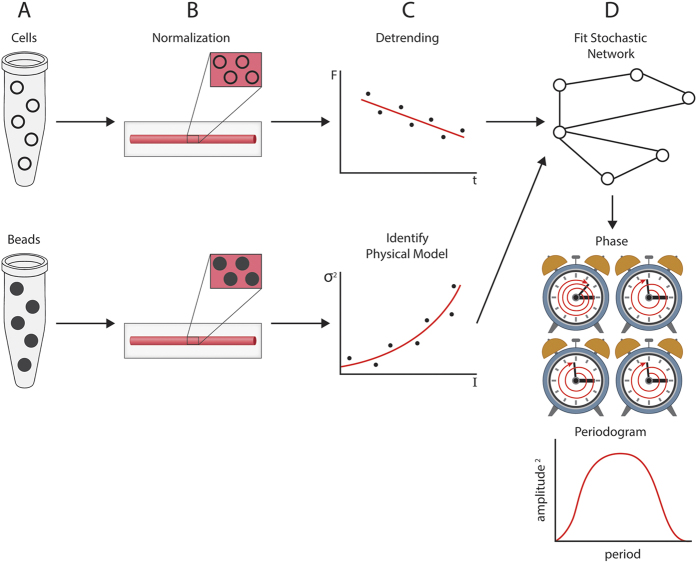
Universal system of measurement on single cells illustrated on single cell oscillators. (**A**) Cells are encapsulated within droplets, and as an internal control fluorescently doped beads are also encapsulated in droplets. (**B**) Cells and beads are immobilized in a capillary tube and viewed over 10 days under a light microscope. The fluorescent signals from cells and beads are normalized by background measurements on Rhodamine B. The variation over time in cell fluorescence captures intracellular stochastic variation; the variation over time in bead fluorescence captures experimental detection noise. (**C**) Normalized fluorescent measurements (**F**) on cells over time (t) are detrended with a moving average. The fluorescence of beads are used to specify a quadratic dependence of signal variance (*σ*^2^) in fluorescence on intensity of light (I) applied to excite the beads to specify a physical model of fluorescent signal detection. (**D**) The normalized and detrended temporal traces on each cell together with the specified physical model for signal detection noise are used to fit a stochastic network. The result is the determination of the amplitude and period in the periodogram for each cell as well as the phase of each single cell oscillator. Phase is defined as the number of cycles completed by an oscillator in a fixed period of time. Four such oscillators are each represented by a clock. One oscillator has completed 4 cycles, and the remaining oscillators have completed only three cycles.

Such a universal system of measurement is proposed here (Fig. 8). Single cell measurements are made here by microfluidics but coupled to doped bead experiments to capture the fluorescent detection noise (Supplement S2). These two sources of error are each propagated through the analysis pipeline for: (1) normalization of the fluorescent signal against Rhodamine B (the internal standard); (2) identification of a physical model for the fluorescent detection; (3) detrending fluorescent measurements due to photobleaching and other sources of variation; (4) propagation of the sources of error to the summary statistics, in this case to the period, phase, and amplitude of the oscillatory system. The periodogram is a standard way to capture period and amplitude (Fig. 2D)^66^ of an oscillator – its ordinate gives the contribution of different sinusoids with frequency f_l_ (=l/T) or period T_l_ contributing to the oscillator. Alternatives to the periodogram exist^67^. The phase in particular has been an elusive^44^ or neglected^39^ quantity. The approach to phase adopted here is one motivated by considering a number of different stochastic clocks (Fig. 8) each with a slightly different measure of time. If these clocks were “in phase”, they would complete the same number of cycles in a fixed period of time, like the wheels on a car. Consider a fixed interval of time with reference to an ideal clock. The phase of a particular stochastic clock is then defined as the number of cycles completed in the fixed interval of ideal time. This is the notion of phase used here (Fig. 3) and introduced over three hundred years ago in the study of synchronization of coupled pendulums^68^. The computation of phase from the Hilbert transform has been in use since the early part of the twentieth century^69^. This notion of phase has been extensively used in synchronization problems in both a physical^44^ and biological context^70^. In this proposed universal system of measurement applied to other problems, the details will vary. There may be additional sources of error, such as intracellular variation^64^. There may be alternate relevant summary statistics, such as fold variation^71^ under two conditions for other processes, such as carbon metabolism^64^, but the structure of the measurement system will be same.

### Summary

Most measurements on the biological clock are made macroscopically on >10^7^ cells. We illustrate a universal system of measurement on single cells. Oscillators in one cell can be measured by microfluidics. Single cells of *N. crassa* are shown to have cell-autonomous circadian oscillators in a fungal system with a biological clock well understood macroscopically. A stochastic mechanism for how the clock functions at the single cell level has been identified and tested that involves transcriptional bursting of *frq* and *ccg-2 by WCC*. At least 94% of the periodogram variation in oscillators from cell to cell is stochastic, a new result for the fungal circadian field, but oscillators of different cells communicate and synchronize to overcome this stochastic asynchrony. This cell-to-cell synchronization has a genetic basis because the synchronization surface varies with genotype.

## Materials and Methods

### Strains and Media

Strain MFNC9^26^ (Fungal Genetics Stock Center #10626) was used in 3% sorbose, 1 M Sorbitol, 0.0125% glucose, 0.0125% fructose, 0.3 mg/ml sodium formate, 1X Vogels Media with recommended Biotin and trace element supplements^72^, as modified from Lindgren^28^. The strains *prd-4* Δ*,ccg-2p:mCherry* and *frq* Δ*, ccg-2p:mCherry* were generated by a cross between a deletion construct^73^ and MFNC9 on cornmeal crossing medium^72^.

### Microfluidics device design

Microfluidic device for cell encapsulation uses a flow-focusing geometry to generate droplets^74^. A mask of the device pattern was designed using AutoCAD 2008 (Autodesk Inc., San Rafael, CA) and printed by a commercial photo-plotting company (CAD/Art Services Inc., Bandon, OR). The prototype polydimethylsiloxane (PDMS) microfluidic device was fabricated through a standard soft-lithography approach and attached to a PDMS substrate^75^.

### Single cell measurements with microfluidics device

A mixture of fluorinated oil FC-40 (Sigma-Aldrich, St. Louis, MO) containing 5.0% w/w of 008 surfactant (RAN Biotechnologies, Inc., Billerica, MA Beverly, MA) was used as the continuous phase to separate the cell suspension into droplets in the microfluidics device. The number of cells per droplet was controlled by the flow rates or the concentration of cell suspensions. The droplets were then collected in a capillary tube with 50 μm in depth (W5010, VitroCom, Mountain Lakes, NJ) for single cell measurement. 13 μL/min was used as continuous phase flow rate and 0.5 μL/min was used as cell suspension flow rate. Cell concentration of 3 * 10 ^ 7 cells/ml was used.

### Imaging

A CCD camera (AxioCam HRm, Carl Zeiss Microscopy, LLC, Thornwood, NY) was used to record the fluorescence intensity of the cells through a microscope (Imager. A1, Carl Zeiss Microscopy, LLC, Thornwood, NY) with a motorized x-y stage (Mechanical stage 75 × 50 R, Carl Zeiss Microscopy, LLC, Thornwood, NY) in a dark room. Images were taken every 30 minutes with an exposure time of 300 ms controlled by a shutter (VS25S2ZM1R1-21, Vincent Associates, Rochester, NY). The excitation light from a mercury lamp (X-Cite 120Q, Dynamics Group Inc., Ramsey, MN) was guided through a filter set (Filter Set 43 HE, Carl Zeiss Microscopy, LLC, Thornwood, NY). A MATLAB routine was developed to sort droplets by the number of cells in them and to track the fluorescence intensity of each cell automatically. A quality control filter code was used to filter out those cells that are improperly tracked. Dark current corrections, bias corrections and flat field corrections were done to all images before tracking the fluorescence intensity of cells.

### Analysis workflow/pipeline of single cell trajectories

In order to calculate the periodograms displayed in Fig. 2C, a few data cleaning steps were performed, as described in Supplement S2. First, the fluorescence time series were divided by a Rhodamine B intensity time series which was gathered concurrently with the cell fluorescence tracking, in order to correct for fluctuations in the microscope’s excitation intensity. Next, we implemented a 24-hour moving average^76^ to remove nonstationarities on large time scales. The resulting individual-cell periodograms were then normalized for further analysis in ensemble fitting. (See Supplement S2 below). A surface of these individual-cell periodograms (Fig. 2C) was created and then averaged together (Fig. 2D).

### Automated cell counting and sizing

Cell counting and sizing was carried out with the Cellometer Auto 2000 (Nexcelom, Inc., Lawrence, MA)^29^. Bright field was set off. There are two fluorescent channels in imaging with exposure times of 500 milliseconds (acridine orange) and 3 seconds (propidium iodide). Both channels used the same parameters for imaging: (1) cell diameter of 4–20 μm; (2) roundness of 0.1; (3) contrast of 0.4; (4) Decluster on with edge factor of 0.5 and Th factor of 1.0; (5) sensitivity of 1.00; (6) uniformity of 150; and (7) fluorescence threshold of 15.

### Ensemble method for fitting a stochastic network model of the clock

The figure of merit in Fig. 2D involved a comparison of the observed, normalized periodograms, 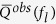, averaged over *N*_*c*_ = 868 single cells, with the average of expected normalized periodogram, 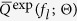, generated under the stochastic model with model parameter variable vector Θ, through a chi-squared criterion:


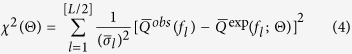


Here, the sum is over the observable frequencies, 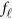, indexed by 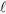, and [*L*/2] = *L*/2, or [*L*/2] = (*L* − 1)/2 for even or odd *L*, respectively. The precise definitions of 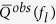 and 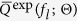 are given in Supplement S2. The 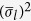 in Eq. 4 is the total variance of 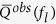 at frequency 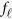 over the sample of *N*_*c*_ = 868 singleton cells in Fig 2. It contains variance contributions generated by both random experimental observation error, *i.e.*, by the fluorescent signal detection process, as well as variance contribution due to the stochasticity of the observed intra-cellular protein concentration. Supplement S2 gives separate estimates for both of these two variance contributions and it also shows how to correct for the bias in the periodogram, caused by the experimental observation variance.

This criterion in Eq. 4 is used to sample and average over the model parameter (Θ−) space by Markov Chain Monte Carlo (MCMC), as described in Supplement S2. Each proposed update of the model parameter vector, Θ, in the MCMC random walk involves generating 1024 trajectories to calculate the expected periodogram under the model, and this step is implemented on GPUs^77^. The result is an ensemble^78^ of stochastic models, Θ, fitting the average periodogram observed on the *N*_*c*_ = 868 cells.

### Propagation of errors to the Periodogram for single cell measurements

The fluorescence detector noise contribution to the “bare” (un-normalized) periodogram variance at frequency *f*_l_ was calculated with


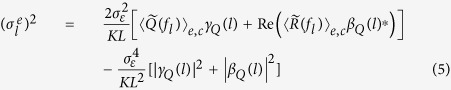


where 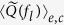 and 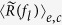 are, respectively, the average bare periodogram and average squared Fourier transform of the observed Rhodamine B-normalized, de-trended protein fluorescence time-series over *K* = 1591 singleton cells. The 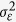 is the variance of this fluorescence signal due to detector noise, averaged across all observation times *t*_*j*_ and across all cells. The quantities *γ*_*Q*_(l) and *β*_*Q*_(l) are functions of the weights used in the moving-average de-trending of cell trajectories. The expectations 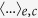 on the RHS are population means over both experimental detection noise and over intra-cellular stochastic randomness. They were replaced by their sample means as a first approximation. The total variance in the Rhodamine B-normalized, de-trended series, (*σ*_l_)^2^, was estimated by bootstrapping 5000 times, the *K* = 1591 cell trajectories, from the replicate experiment in Fig. 5A. The stochastic intra-cell variance (*σ*_l_^c^)^2^ was obtained by subtraction, using (*σ*_l_^c^)^2^ = (*σ*_l_)^2^ − (*σ*_l_^e^)^2^. The proportion of variation in a cell oscillator fluorescent signal attributable to detector noise (*i.e*., experimental error) was then: (*σ*_l_^e^)^2^/(*σ*_l_)^2^ < 6% for all frequencies 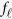. Details of this calculation are found in Supplement S2.

## Additional Information

**How to cite this article**: Deng, Z. *et al*. Synchronizing stochastic circadian oscillators in single cells of *Neurospora crassa*. *Sci. Rep.* **6**, 35828; doi: 10.1038/srep35828 (2016).

**Publisher’s note:** Springer Nature remains neutral with regard to jurisdictional claims in published maps and institutional affiliations.

## Supplementary Material

Supplementary Information

Supplementary video S1

## Figures and Tables

**Table 1:** Analysis of Variance (ANOVA) of Periodogram for 868 singleton cell trajectories for 170 time points.

Source	Degrees of Freedom (df)	Sums of Squares (SS)	Estimated Mean Square (EMS)	F-value
Model Frequency 0.0353 h^−1^ (period = 28 h)	2	0.045225	0.0226125	7.67***
Model Frequency 0.0471 h^−1^ (period = 21 h)	2	0.150372	0.075186	25.51***
Model Frequency 0.0575 (period = 17 h)	2	0.160925	0.0804625	27.30***
Model Frequency 0.0706 h^−1^ (period = 14 h)	2	0.104017	0.0520085	17.64***
Model Frequency 0.0824 h^−1^ (period = 12 h)	2	0.067837	0.0339185	11.51***
Error	160	0.471624	0.00294765	
Total	170	1		

The model contribution to the Sums of Squares (SS) are from the fitted stochastic network model of the clock. For simplicity of interpretation each SS is scaled so that their sum is 1. Each frequency (or period) in the periodogram contributes 2 degrees of freedom to the model sums of squares. SS_model/SS_total x 100 = 53%. The estimated mean square (EMS) is a sum of squares (SS) divided by its degrees of freedom. The F-value is the ratio of an EMS of the model to the EMS calculated from the SS of the error = stochastic error + measurement error. The contribution of each frequency to the minimized sums of squares is significant at P < 0.001 (***).

**Table 2:** Analysis of Variance (ANOVA) of fluorescence of the *ccg-2* promoter between and within droplets is used to estimate the intraclass correlation ρ.

Source	degrees of freedom (df)	Sums of Squares (SS)	Estimated Mean Square (EMS)	Expectation of EMS
Between droplets	n − 1	SS_B_ = *a* ∑_ι_(XBAR_i_ − XBAR)^2^	SS_B_/(n − 1)	*σ*^2^ + (a − 1)ρ*σ*^2^
Within droplets	n(a − 1)	SS_W_ = ∑_i_∑_j_(X_ij_ − XBAR_i_)^2^	SS_W_/n(a − 1)	(1 − ρ)*σ*^2^
Corrected total	na − 1	SS_T_ = ∑_i_∑_j_(X_ij_ − XBAR)^2^		

The one-factor model is a balanced variance components model with cells grouped by droplets^45^. The number of cells in a droplet is *a*; the number of droplets is n. The variance in a single fluorescent measurement is *σ*^2^, and the intraclass correlation is ρ. The intraclass correlation is a measure of fraction of total variation (SS_T_) within droplets as well as the correlation between fluorescence measurements on different cells within the same droplet. The statistics XBAR_i_ and XBAR are the mean fluorescence in droplet i and over all droplets. The estimated mean square (EMS) is a sum of squares (SS) divided by its degrees of freedom. The EMS estimates its expectation in the last column. Setting the EMS equal to their expectations allows the solution for ρ.

**Table 3:** The mean period of single cell oscillators within a droplet increases significantly with the number of cells per droplet at two major frequencies in the periodogram, and the variance in period among single cell oscillators within a droplet decreases significantly with the number of cells per droplet at two major frequencies in the periodogram.

Number of Cells/Droplet (a)	Primary harmonic		Secondary harmonic	
	Mean of Period	Variance of Period	Mean of Period	Variance of Period
1	16.357	57.06	9.077	56.095
2	16.608	52.225	9.3168	53.431
3	16.18	53.93	9.3788	52.724
4	16.426	53.351	10.025	52.699
5	17.068	50.007	10.468	52.338
6	16.504	49.729	10.267	49.443
7	16.324	47.549	10.115	47.244
8	16.23	51.906	9.708	47.59
9	16.537	51.911	10.065	49.642
10	16.701	50.377	10.539	47.125
11	16.206	55.129	10.424	48.35
12	17.034	41.147	10.86	44.434
13	16.388	46.923	10.171	47.247
14	16.768	45.904	10.725	46.066
15	17.214	42.897	11.502	42.188
16	17.146	46.499	10.893	53.131
17	17.445	37.217	11.106	42.044
18	16.879	53.687	9.6857	52.153
19	16.853	49.419	10.92	40.386
20	16.129	39.879	10.628	24.815

Each single cell oscillator was examined at two frequencies in the periodogram, the frequency with the highest peak (primary harmonic) and the frequency with the second highest peak (secondary harmonic). Each cell’s periods were computed by determining the maximum in the periodogram and second largest maximum in the periodogram on each cell trajectory after normalization and detrending. With respect to the highest peak in the periodogram (labeled primary harmonic below) the Spearman rank correlation (r_s_) of average period of cells with the number of cells per droplet (a) is r_s_ = 0.3669 (P < 0.10), and the Spearman rank correlation (r_s_) of variance of period within a droplet with the number of cells per droplet is r_s_ = −0.6316 (P < 0.01)^80^. With respect to the second highest peak in the periodogram (labeled secondary harmonic) the Spearman rank correlation (r_s_) of average period of cells with the number of cells per droplet (a) is r_s_ = 0.7083 (P < 0.005), and the Spearman rank correlation (r_s_) of variance of period within a droplet with the number of cells per droplet is r_s_ = −0.7083 (P < 0.005)^80^.

**Table 4:** The intraclass correlation surface (ICC) for *prd-4* Δ*,ccg-2p:mCherry* is significantly different from that of MFNC9.

y	x	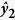	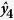	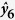	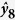	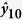	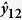	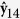	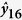	R^2^
MFNC9-rep2	MFNC9-rep1	0.29	0.36	0.49	0.49	0.51	0.56	0.44	0.31	0.70
2 × Standard error (SE)		0.006	0.006	0.004	0.004	0.004	0.004	0.004	0.006	
*prd-4*^Δ^	MFNC9-rep1	0.40	0.48	0.82	0.70	0.72	0.49	0.53	0.57	0.61
2 × SE		0.016	0.016	0.010	0.010	0.010	0.014	0.010	0.018	
*frq*^*Δ*^	MFNC9-rep1	0.37	0.36	0.60	0.50	0.40	0.56	0.50	0.29	0.84
2 × SE		0.012	0.010	0.006	0.006	0.006	0.008	0.006	0.012	

To assess this one ICC surface (y) was regressed on another ICC surface (x), and the following relation y = mx + b_*a*_ was fit by least squares to the plots in Fig. 7. In each fit a constant slope (m) and intercept (b_*a*_), which varies with the number of cells per droplet (*a*), w*a*s assumed. The regression R^2^ summarizes the fraction of variation in the data captured by the linear regression of y on x. The mean ICC values are reported for droplets of even sizes (a) from 2 to 16 cells per droplet.
