# Supplementary material for: Synchronizing stochastic circadian oscillators in single cells of Neurospora crassa
Source: Sci Rep. 2016 Oct 27;6:35828. doi: 10.1038/srep35828 (PMC5082370; doi:10.1038/srep35828)
Supplement: Supplementary Information [file srep35828-s1.pdf]

Supplementary Materials for

Synchronizing stochastic circadian oscillators in single cells of *Neurospora crassa*

Zhaojie Deng, Sam Arsenault, Cristian Caranica, James Griffith, Taotao Zhu, Ahmad Al-Omari, Heinz-Bernd Schüttler, Jonathan Arnold, and Leidong Mao

correspondence to: arnold@uga.edu and mao@uga.edu

This PDF file includes:

Supplementary Text with Table S1, Figs. S1, S2, S3, S4, S5, S6, S7, S8, S9, and caption for Video S1

Other Supplementary Materials for this manuscript includes the following:

Video S1

Supplementary Text

S1 Experimental protocol for single cell measurements on the biological oscillator of *Neurospora crassa*

- *N.crassa* strains, MFNC9 and MFNC30, growth

1.Inoculate a slant or multiple slants (depending on the growth characteristics of the desired strain) of growth media[1]with *N. crassa* conidia from a frozen culture.

2.Then put the cells at 30 °C in darkness to grow for about 48 h and then put them under light conditions on the bench top at room temperature. Keep the cells there for a minimum of 24 h, even 3-4 days to achieve maximum growth.

3.Then collect the conidia by suspension with sterile water (or other desired media). Determine the amount of suspension solution to be used by the growth and desired conidial concentration.

4.Filter the conidial suspension through a funnel containing a plug of sterile cotton into a sterile flask. Then pour the filtered conidial suspension into a 50 ml screw cap tube for storage.

5.Take a 20 µl sample of the conidial suspension and measure it on the cellometer (Nexcelom Cellometer Auto 2000, Nexcelom Bioscience LLC., Lawrence, MA) to determine the concentration of the conidia. (This measurement can also be taken with a haemocytometer under a light microscope.)

6.Then either dilute (with additional liquid) or concentrated (by centrifugation) the conidia to obtain the needed concentration.

- PDMS device preparation and surface treatment

Fabricate the prototype polydimethylsiloxane (PDMS) microfluidic device for droplet generation through a standard soft-lithography approach and attach it to a glass slide[2]. The workflow of making the device is as follows.

1.Spin coat a silicon wafer of diameter 100 mm (Addison Engineering Inc., San Jose, CA) with negative photoresist, SU-8 2025 (MicroChem Corp., Newton, MA).

2.Print the pattern of the photomask on the silicon wafer using a MA6 mask aligner (SUSS MicroTec Inc., Sunnyvale, CA) under a UV light source of wavelength 365 nm.

3.Develop the silicon wafer by submerging it in SU-8 developer (MicroChem Corp., Newton, MA) for several minutes.

4.Mix the pre-polymer Sylgard 184 (Dow Corning Corp., Midland, MI) with a cross-linker in a ratio of 10:1 w/w and then pour them onto the patterned silicon wafer and degas the mixture in a desiccator.

5.Put the mixture at 80 °C for 2 h for curing.

6.After curing, cut the PDMS layer and peel it off the wafer. Punch Inlets and outlets using a biopsy punch (Milte, Inc., York, PA) with an outer diameter of 1 mm.

7.Measure the thickness of the device by a profilometer (Dektak 150, Veeco Instruments Inc., Chadds Ford, PA). Normally, the thickness of the device is 50 µm.

8.Before attaching to a glass slide, treat the channel surface with air plasma (PDC-32G plasma cleaner, Harrick Plasma, Ithaca, NY) at 11.2 Pa O_2 partial pressure with 18 W power for 2 min.

9.After attachment, put the device in the oven at 80 °C for 8 h.

10.Afterwards put the device into a petri dish. Put the petri dish in a desiccator in a fume hood. Pipette 25 μL 1H,1H,2H,2H-Perfluorooctyltriethoxysilane (Sigma-Aldrich, St. Louis, MO) into the petri dish and let them surround the device.

- <u>Generate droplets with encapsulated cells and droplet collection</u>

1.Dilute the original cell suspension to the needed concentration for encapsulating single-cells/multiple-cells in droplets. Use percoll as a dispersant to prevent the cells from settling down in the syringe during cell encapsulation

2.Insert ~2 cm of PE tubing into the two inlets of the device. Flush the device with DI water for the outlet to get rip of the PDMS debris.

3.Load the continuous phase into a 1 mL syringe. The continuous phase is a mixture of fluorinated oil FC-40 with 5.0 wt% of 008 fluorosurfactant.

4.Load the final cell suspension into a 1 mL syringe.

5.Connect the loaded syringes to the inlets of the devices with PE tubing and the tygon tubing.

6.Put the devices onto the stage of the microscope and secure the syringes to the slot of syringe pump. Set the flow rate of the continuous phase to be 13 uL/min and that of the dispersed phase to be 0.5 uL/min. and then run the syringe pump.

7.Connect the outlet of the device with a 1.5 mL conical tube using a PE tubing to collect the droplets in the conical tube.

8.After enough droplets are collected in the conical tube. Use a syringe to take out the redundant carrier oil (the fluorinated oil). Then use a capillary tube to retrieve the droplets for observation by submerging one end of the capillary tube in the droplets layer in the conical tube.

9.Put the capillary tube onto a clean glass slide and seal the tube with Epoxy glue.

10. To synchronize the cells, put the glass slide with capillary tube under the light source for 26 h.

- <u>Image processing</u>

1.Correction of CCD Imaging Imperfections

1.1 Dark current correction: Take ten images with the same exposure time as the time-lapse experiment before each experiment but with the shutter closed. Average these images to create a dark current image. Subtract from each fluorescence image, the dark current image.

1.2 Bias correction: Take a few of images with zero exposure time and with the shutter closed. Average these images to create a bias image. Subtract from each fluorescence image (corrected for dark current), the bias image.

1.3Flat field correction: Take a couple of images with quite short exposure time of uniformly illuminated screen/field of view. A Rhodamine B solution with a concentration of 6.6 μM in a 35 mm culture dish was used as the uniformly illuminated screen. Average these images to create a flat field image. Obtain the average pixel value within the (dark/bias corrected) flat field image (call it α). Then, for each fluorescence image (dark/bias corrected), multiply all pixels by α and then divide (pixelwise) by the flat field image. Flat field image should be created each time optical properties of the illumination and lens system are changed. For example, a different flat field image is used for different magnifications.

2.Fluorescence intensity data extraction
We wrote a Matlab routine to sort droplets according to the number of cells in them and to track the fluorescence intensity of individual cell frame by frame. Locations of cells are determined by finding a local maximum in fluorescence intensity. The sorting is based on the difference of distance between cells and droplet and the radius of the droplet. The tracking is mainly based on the minimum distance between cells in consecutive frames. The quality control filter to filter out cells that are not linked property is based on comparing values of the extracted fluorescence intensity in consecutive time for one cell and between cells. The quality control filter marks the cells that are not linked properly as bad cells and they are not considered later in the analysis.

Additional controls recommended are an examination of a time lapse video S1 for detecting germination, fusion of cells, and cell division. In addition we selected ~200 singletons and monitored surface area over time for germination and cell division (10 are shown in Fig. S5). In addition, we plotted ~200 random pairs of cells and tracked them over time to see if there was any change in distance between them that might be evidence of cell fusion (10 are shown in Fig. S5).

We also recommend an examination of a phase histogram of single cells (Fig. 3) to validate that the phase histogram is unimodal. Bimodality might arise if cells were not all synchronized at the same time. A dip test was performed ($P = 2.2 \times 10^{-16}$)[3]. The fact that a tiny dip of the same size also occurs in the stochastic simulation (Fig. 3D) may suggest that the dip test does not capture the intracellular stochastic variation in the data and that we may need to speed up encapsulation to less than 20 m, the current time taken for the encapsulation process. A third control is to substitute doped beads for cells and observe the beads over 10 days in the capillary tube. The periodogram of single beads should be computed (Fig. 3). We found a maximum in the periodogram at 20 h of about 0.03 (more than 4-fold less than the maximum in periodogram in Fig 2D), which is explainable by the 1 ^{o}C fluctuation in temperature occurring diurnally during the microfluidics experiment. A fourth control is to examine the period of ensembles of oscillators in various strains in race tubes to confirm that the period of each ensemble is the same as that of individual cells measured by microfluidics. A final control is to examine race tubes exposed to a flash on a iPhone every 30 minutes at the same intensity as the exposure of single cells to light every 30 minutes to observe fluorescence. We confirmed no change in the clock phase and period in race tubes relative to tubes without a flash exposure every 30 m. The banding with the light show some irregularity compared with those race tubes in total darkness, but the light exposure was longer (~0.5 seconds) in Fig. S6.

S2 MCMC procedure developed for fitting a stochastic genetic network

- Stochastic Model of the Clock's Intra-Cellular Reaction Kinetics

The time evolution of a well-stirred chemical system, such as the biological clock, is usually described by a system of ordinary differential equations (ODEs) that show the evolution of molecular numbers of species in the system as a continuous, deterministic process[4]. The problem with this approach is that molecular numbers are given by whole numbers, and when they change, they always do so by discrete amounts. If molecular numbers of some species are small or if the dynamics of the chemical system is

susceptible to noise[5], then a system of ODEs is unlikely to describe completely the dynamics of such a system . We need a discrete, stochastic model to describe the noisy behavior of the system, and we need a stochastic model to describe how chemical reactions occur as discrete events at the molecular level. Such a model, and an algorithm to solve it, was devised by Gillespie[6].

- Gillespie algorithm

We consider a system of N_S chemical species $\{s_1, s_2, ... s_{N_S}\}$ interacting through N_R chemical reactions $\{r_1, r_2, ... r_{N_R}\}$. The system is assumed to be confined to a constant volume. The number of molecules of species s_n at time t, denoted by $X_n(t)$, is an integer-valued, piecewise constant function of time. $X_n(t)$ undergoes discontinuous jumps at random points in time, whenever a reaction event occurs which consumes or produces molecule(s) of species s_n. To simulate the evolution of the state vector, $X(t) = \{X_1(t), X_2(t), ..., X_{N_S}(t)\}$, we need to be able to calculate two things: how much time will pass until the next reaction event occurs and which reaction will occur at that time.

Each reaction r_j is characterized by two quantities. The first is its state-change vector $\upsilon_j = (\upsilon_{1,j}, \upsilon_{2,j}, ..., \upsilon_{N_S,j})$, where $\upsilon_{l,j}$ is the change in the molecular number of species s_l, caused by one r_j -reaction event. These state-change vectors are defined by the topology of the reaction network[4], as summarized graphically in Fig. 4A. If the system is in state $X(t) = x$ at time t and reaction r_j occurs, then the system instantaneously changes to state $x + \upsilon_j$. The second quantity characterizing r_j is its propensity function a_j. Given a state $X(t) = x$, $a_j(x)dt$ is the probability that one reaction R_j occurs in the next infinitesimal time interval $[t, t+dt)$. The following assumptions, based on physical principles, allow us to determine the distribution of the next reaction time and the probability that r_j is the next reaction.

(1) $a_j(x)dt$ =probability that reaction r_j takes place in the infinitesimal time interval $[t, t+dt)$.

(2) $a_j(x) = k_j C_j(x)$, where k_j=reaction rate coefficient of r_j and $C_j(x)$=number of possible ways r_j can occur given system state x. (This is a mass action assumption used in the working model of the clock now supported by over 30,000 data points[7]). The rate constants k_j are listed in Fig. 4A under the no coupling assumption.

For instance, $C_j(x)$ is 1, $X_l(t)$, $X_l(t)X_m(t)$ and $\frac{1}{2}X_l(t)(X_l(t)-1)$ for zeroth-order, unimolecular, hetero-bimolecular and homo-bimolecular reactions, respectively,

involving species s_l and/or s_m for given the state $x \equiv \{X_1(t), X_2(t), ..., X_{N_S}(t)\}$ prior to the reaction event.

For a given model parameter set, i.e., given rate coefficients and initial conditions, we generate over 1000 random trajectories of the stochastic clock model using the Gillespie Algorithm[6]. Each trajectory starts from the given set of initial molecular numbers for all species, $X(0)$, at time $t = 0$. Random-length time steps, from one reaction event to the next, are then performed, based upon foregoing assumption (1), that the time until the next reaction, τ, is exponentially distributed with a rate

$$a_0\big(X(t)\big) = \sum_{k=1}^{N_R} a_k\big(X(t)\big) \tag{S1}$$

where t is either the time of the most recent reaction event, or else $t = 0$. Thus, a random τ is drawn from that exponential distribution. The probability that this next reaction is r_j, is given by

$$a_j(X(t)) / a_0(X(t)) \tag{S2}$$

Thus, to decide *which* reaction, r_j, takes place next, we choose a random number j between 1 and N_R according to these probabilities. Namely, we draw a uniform random number u between 0 and 1 and find *that* index value j for which

$$\sum_{k=1}^{j-1} a_k(X(t)) < u\, a_0(X(t)) \le \sum_{k=1}^{j} a_k(X(t)) . \tag{S3}$$

Then, the reaction that occurs next is r_j. Time t is then advanced to that next reaction event by a time step of length τ.The trajectory is terminated when the last reaction event time, t ,exceeds the total model simulation time duration, T. In summary, the Gillespie trajectory generation algorithm works as follows:

1) Initialize: set initial number of molecules, reaction rates and set *t*=0
2) Calculate the propensity functions $a_j(X(t))$, for all $j = 1, ..., N_R$
3) Choose τ according to the exponential distribution with parameter
   $a_0\big(X(t)\big) = a_1\big(X(t)\big) + ... + a_{N_R}\big(X(t)\big)$,
   i.e., draw $r \sim Unif(0,1)$ and take $\tau = -\log(r) / a_0(X(t))$.
4) Choose j as in Eq. (S3) above and update the state of the system from $X(t)$ to $X(t+\tau) = X(t) + \upsilon_k$, to reflect the execution of reaction r_j. Set $t = t + \tau$.
5) Go back to step 2 if $t < T$, else stop.

To estimate stochastic model averages, such as a the stochastic mean of a periodogram (see below), a large number, $K^{(Model)}$, of random Gillespie trajectories are generated , for a given model parameter vector, denoted by $\Theta \equiv \{X_1(0), X_2(0), ... X_{N_S}(0), k_1, k_2, ... k_{N_R}, \phi\}$,

typically with $K^{(Model)} \geq 1000$. Here, k_j denotes the rate coefficient of reaction r_j and $\sqrt{\phi}$ is the (generally unknown) unit conversion factor from the fluorescent protein molecule count into the corresponding normalized and de-trended fluorescent photon count signal, as measured experimentally by the CCD and explained below. The mean is then taken over these $K^{(Model)}$ simulated trajectories, *e.g.*, for the mean bare periodogram, as follows:

$$Q^{\exp}(f_\ell;\Theta) = \frac{1}{K^{(\text{Model})}} \sum_{k=1}^{K^{(\text{Model})}} Q_k^{\exp}(f_\ell;\Theta) \tag{S4}$$

and the periodogram (power spectrum), of the kth Gillespie trajectory is given by

$$Q_k^{\exp}(f_\ell;\Theta) = \phi \left| \frac{1}{L} \sum_{j=1}^{L} \exp(-2\pi i\, f_\ell\, t_j)\; X_{n_{\text{obs}},k}(t_j;\Theta) \right|^2 \tag{S5}$$

Here, $X_{n_{\text{obs}},k}(t_j;\Theta)$ is the molecule count of the observable (*i.e,* fluorescent) protein species, $s_{n_{\text{obs}}}$, for the kth Gillespie trajectory at time $t_j = (j-1)T/L$. The frequencies in Eq.(S5) are $f_\ell = \ell/T$. Respective integer indices are $j = 1,2,...\ L$ and $\ell = 0,1,...\ [L/2]$ where $[x]$ denotes the maximum integer not exceeding x; and i is the imaginary unit: $i^2 = -1$. Hence, L is the total number of equidistant observation time points, t_j, of spacing T/L, corresponding to a sampling rate of $f_{\text{samp}} = L/T$ with a value of f_{samp} = 2 observations per hour in all our single-cell experiments.

- Fluorescent Signal Data Normalization

Several corrective data transformations and a detailed noise analysis must be performed with the observed single-cell fluorescence time series raw data, before they can be Fourier analyzed to obtain periodograms which can then be meaningfully compared to the stochastic model predictions. The basic detection method for time-varying single-cell protein concentrations is to record the fluorescent single-cell signal, generated by the protein molecules upon illuminating the cell with radiation from a shorter-wavelength light source. Let $S_k(t_j)$ denote the mean fluorescent photon count signal produced by singleton cell $\# k$ during the CCD exposure time interval at observation time t_j, measured in units of the CCD camera read-out. This "mean signal" is assumed to be free of detection noise and proportional to the number of fluorescent protein molecules in the cell, denoted by $X_k(t_j)$ here. [The species index n_{obs} in $X_k(t_j)$ is dropped here, since only this one fluorescent protein species is observed in the experiments.] However, $S_k(t_j)$ is also proportional to the mean count of illumination photons, $I(t_j)$, incident upon the cell from the lamp during CCD exposure at observation time t_j. Hence,

$$S_k(t_j) = a_C I(t_j) X_k(t_j) \tag{S6}$$

The proportionality constant a_C is a property of the fluorescent protein molecule, *i.e.*, a_C depends *only* on the fluorescence properties of a single fluorescent protein molecule in its

intra-cellular environment, but is assumed to be the same across all cells at all observation times. The time and cell indices, respectively, are $j = 1,...,L$ and $k = 1,...,K$. Here, L is again the total number of equidistant observation time points $t_j = (j-1)T/(L-1)$, and K is the total number of cells. For the experimental analysis and model ensemble simulations reported here we used T=85h, $L = 171$, and $K = 1591$, unless stated otherwise, corresponding to an observation time interval $\left|t_{j+1} - t_j\right| = 0.5$h.

Since the illumination intensity produced by the lamp can vary with time over the 200+ hour duration of the experiment, a reference measurement is made, almost at the same time as each fluorescent cell observation, to also detect the fluorescent intensity, Z, from a Rhodamine B (RB) sample illuminated with the same lamp, and hence also proportional to the illumination photon count, I:

$$Z\left(t_j\right) = a_R I\left(t_j\right) \tag{S7}$$

The proportionality constant, a_R, is a property solely of the RB sample and assumed to be constant in time. Hence, the time-dependence of the single-cell signal, S, due to the time-varying illumination can be cancelled out by normalizing the single-cell signal, S, to the reference intensity, Z, *i.e.,* by taking

$$Y_k\left(t_j\right) = \frac{S_k\left(t_j\right)}{Z\left(t_j\right)} = \frac{a_C}{a_R} X_k\left(t_j\right) \tag{S8}$$

which is proportional *only* to the fluorescent protein count in cell $\#k$ at time t_j. The foregoing results now need to be extended to allow for the presence of detection noise.

Note in passing that the ratio a_C / a_R in Eq.(S8) is related to the periodogram unit conversion factor ϕ in Eq. (S5) by: $\phi = (a_C / a_R)^2$. Since that ratio is unknown, so is ϕ. Hence, ϕ is treated as an unknown, adjustable model parameter, along with all other stochastic model parameter variables in $\Theta \equiv \left\{X_1(0), X_2(0),...X_{N_S}(0), k_1, k_2,...k_{N_R}, \phi\right\}$.

- Intra-Cell Stochasticity and Detection Noise in Observed Signals

The cellular fluorescent signals, $S_k\left(t_j\right)$, in Eq. (S6) are random variables due to the stochasticity of the intra-cellular chemical processes. They reflect the stochastic nature of the underlying chemical reaction network, *via* the fluorescent protein molecule counts, $X_k\left(t_j\right)$, in Eq. (S6). By definition, $S_k\left(t_j\right)$, does not contain any randomness from the experimental detection process. The detection process adds further randomness in producing the CCD detector output, denoted by $\tilde{S}$ and consisting of the stochastic detector input signal, S, superimposed with a detection noise term, ξ:

$$\tilde{S}_k\left(t_j\right) = S_k\left(t_j\right) + \xi_k\left(t_j\right) \ . \tag{S9}$$

In the following, all cell-related fluorescence signal and respective periodogram quantities containing random contributions from experimental detection noise will be

identified with a tilde overscript, such as $\tilde{S}_k(t_j)$, as well as quantities such as $\tilde{Y}_k(t_j)$, $\tilde{V}_k(t_j)$, $\tilde{Q}^{\text{obs}}(f_l)$, $\tilde{Q}(f_l)$, $\tilde{Q}_k(f_l)$, $\tilde{F}_k(f_l)$ *etc.*, defined below. Corresponding quantities that would be observed (hypothetically) in the absence of any experimental detection noise are denoted without the tilde overscript, such as $S_k(t_j)$, $Y_k(t_j)$, $V_k(t_j)$, $Q^{\text{obs}}(f_l)$, $Q(f_l)$, $Q_k(f_l)$, $F_k(f_l)$ *etc.* The latter quantities exhibit randomness only due to the stochasticity of the intra-cellular chemical processes, as reflected in $X_k(t_j)$.

Each detection noise variable, $\xi_k(t_j)$, in Eq. (S9) is assumed to be statistically independent across all cells, k, and all times t_j, with zero mean and a detection noise variance $(\sigma_{\xi,k,j})^2$:

$$\left\langle \xi_k(t_j)\xi_{k'}(t_{j'})\right\rangle_e = (\sigma_{\xi,k,j})^2\,\delta_{k,k'}\delta_{j,j'} \tag{S10}$$

where $\delta_{k,k'}$ and $\delta_{j,j'}$ are Kronecker delta-functions. The bracket $\langle\ldots\rangle_e$ denotes the conditional population average over all possible experimental detection noise configurations, *given* the "clean" detector input signal, $S_k(t_j)$, prior to the addition of detection noise. That is, $\langle\ldots\rangle_e$ is an average over a (hypothetical) infinite number of repeats of the detection process, each repeat detection performed with the same, fixed detector input, $S_k(t_j)$. Thus, Eq. (S10) is *not* averaged over, but conditional upon, the stochastic intra-cellular state trajectories, $X_k(t_j)$, and the resulting noise variances, $(\sigma_{\xi,k,j})^2$, depend on the stochastic detector *input* signal $S_k(t_j)$, given by Eq. (S6).

The detection noise likewise modifies the illumination-normalized pre-detection signal, Y, with a noise term denoted by ε, resulting in an illumination-normalized post-detection signal

$$\tilde{Y}_k(t_j) := \frac{\tilde{S}_k(t_j)}{Z(t_j)} = Y_k(t_j) + \varepsilon_k(t_j) \ , \tag{S11}$$

again with statistically independent noise variables, $\varepsilon_k(t_j) = \xi_k(t_j)/Z(t_j)$, of variance $(\sigma_{\varepsilon,k,j})^2$:

$$\left\langle \varepsilon_k(t_j)\varepsilon_{k'}(t_{j'})\right\rangle_e = (\sigma_{\varepsilon,k,j})^2\,\delta_{k,k'}\delta_{j,j'} \qquad \text{with} \qquad \sigma_{\varepsilon,k,j} = \frac{\sigma_{\xi,k,j}}{Z(t_j)} \, . \tag{S12}$$

We are neglecting the detection noise effect on the reference signal, $Z(t_j)$. The latter is recorded by a large number of pixels, across the entire field of view of the detecting CCD camera and it is also not subject to variability from random focal point excursions. Based on the detection noise analysis described above and below, the reference signal can thus

be assumed to have a much lower detection noise level, as a percentage of signal, than the cell-generated detector output signals $\tilde{S}_k(t_j)$.

Intra-cellular stochasticity and the detection noise *both* contribute, statistically independently, to the random variability of the actually observed CCD detector output signal $\tilde{S}_k(t_j)$. It is necessary to separate and account for both sources of randomness in our modeling, since only the intra-celluar stochasticity, but not the detection noise, is of biological interest. As discussed below, the detection noise also introduces a bias into the periodograms constructed from the observed output signals, $\tilde{S}_k(t_j)$. A separate estimate for the detection noise variance is thus needed to correct for this bias.

There are six sources of detection randomness in single cell measurements that are associated with the fluorescent signal generation and detection from a cell, as follows: (1) the quantum randomness of fluorescent photon generation in the cell; (2) the cell's random focal plane excursions; (3) the quantum randomness of photo-electron generation in the CCD camera; (4) CCD random pixel-to-pixel variations in each pixel's photon-to-electron conversion yield; (5) CCD image pixel summation; (6) CCD bias and dark current subtraction. For the analysis that follows, these six noise sources are assumed to be statistically independent and to add noise successively, with the noisy output signal from one noise source serving as the input signal of the next. Furthermore, we assume, on simple physical grounds, that each of the foregoing six noise sources can be classified as either 0th, 1st or 2nd order, in the sense that the variance of the noise added to the input signal by a 0th, 1st or 2nd order source is proportional to the 0th, 1st or 2nd power of the source's input signal, respectively. Of the foregoing six noise sources, source (6) is 0th order, sources (1) and (3) are 1st order, and sources (2), (4) and (5) are 2nd order. A detailed mathematical noise compounding and propagation analysis then shows that the successive application of any sequence of 0th, 1st or 2nd order detection noise sources to the detector input signals $S_k(t_j)$ can all be lumped into a single "effective" noise source, with statistically independent "effective" noise variables, $\xi_k(t_j)$, whose respective variances depend on the original detection input signal by a simple quadratic relation of the form:

$$\left(\sigma_{\xi,k,j}\right)^2 \equiv \left\langle \xi_k\left(t_j\right)^2 \right\rangle_e = C + A\, S_k\left(t_j\right) + B\, \left(S_k\left(t_j\right)\right)^2 . \qquad \text{(S13)}$$

The coefficients of this quadratic function, A, B and C, can be expressed in terms of the various proportionality coefficients of the underlying individual 0th, 1st or 2nd order sources. These proportionality coefficients can not be estimated from available experiments for each individual source. A, B and C must therefore be determined by a separate "noise calibration" experiment where the intrinsic noise variance of the detector input signal is either zero, or can be quantified separately by other means. The analysis and results of these noise calibration experiments, using samples of fluorescent beads instead of living cells, will be described in the following section below.

To simplify the periodogram noise analysis discussed below, we make the additional assumption that the variances $\left(\sigma_{\xi,k,j}\right)^2$ can be approximated by their population mean over the stochastic intra-cellular molecule count time series, $X_k(t_j)$, as measured by the mean photon count signal $S_k\left(t_j\right)$ in Eq. (S6), *i.e.*, by Eq. (S13):

$$(\sigma_{\xi,k,j})^2 \cong (\sigma_{\xi,j})^2 \equiv C + A\left\langle S_k(t_j)\right\rangle_c + B\left\langle \left(S_k(t_j)\right)^2\right\rangle_c \tag{S14}$$

where $\langle\ldots\rangle_c$ denotes the population mean over the intra-cellular stochastic time series, $X_k(t_j)$. The resulting means $\left\langle S_k(t_j)\right\rangle_c$ and $\left\langle (S_k(t_j))^2\right\rangle_c$, and hence $(\sigma_{\xi,j})^2$, are independent of the cell index *k*, since, by model assumption, the random distribution of the stochastic intra-cellular time series, $X_k(t_j)$, is the same for all cells.

From Eqs. (S9), (S10) and (S13), we then get

$$(\sigma_{\xi,j})^2 = \frac{1}{1+B}\left[C + A\left\langle \tilde{S}_k(t_j)\right\rangle_{e,c} + B\left\langle \left(\tilde{S}_k^2(t_j)\right)^2\right\rangle_{e,c}\right] \tag{S15}$$

where $\langle\ldots\rangle_{e,c} \equiv \left\langle\langle\ldots\rangle_e\right\rangle_c$ denotes the joint population average over detection noise configuration and over intra-cellular stochastic time series variable, $X_k(t_j)$. For large cell sample sizes, *K*, the foregoing population averages can be approximated by sample averages over the actually observed CCD signals from all cells:

$$\left\langle \tilde{S}_k(t_j)\right\rangle_{e,c} \cong \frac{1}{K}\sum_{k=1}^{K}\tilde{S}_k(t_j) \quad \text{and} \quad \left\langle \left(\tilde{S}_k^2(t_j)\right)^2\right\rangle_{e,c} \cong \frac{1}{K}\sum_{k=1}^{K}\left(\tilde{S}_k^2(t_j)\right)^2 . \tag{S16}$$

Eqs. (S16) and (S15) were then used to estimate the detection noise variances $(\sigma_{\xi,k,j})^2$.

- <u>Detection Noise Calibration by Bead Experiments</u>

To quantify the detection noise level in the fluorescent single-cell signals by way of the quadratic signal-to-noise relation, Eq. (S15), we performed a series of noise calibration experiments where the sample of droplet-encapsulated living cells was replaced by a nearly mono-disperse sample of small, droplet-encapsulated, fluorescent beads. The beads were polymer microspheres internally doped with fluorescent dye, Cat. Code: FS06F, Envy Green (Excitation/Emission:525nm/565nm), with mean diameter 9.94 μm and diameter standard deviation 0.76 μm, as provided by the manufacturer. In contrast to the cellular fluorescent signals, the mean fluorescent output signal from each bead is not subject to any intrinsic time-dependent stochastic variability. However, the beads do exhibit time-independent variability in their fluorescent signal output due to a small, known, but non-negligible variance in the bead size distribution. The overall variability in the CCD detector output signal observed in the bead experiment is thus the result of the experimental detection noise and of the bead size variability. The latter can be treated as an added 2nd order noise source, while the six noise sources from the detection process are the same as those in the single-cell experiment. By subtracting out the signal variability caused by the random bead sizes, we can thus extract from the bead

experiments the noise calibration coefficients, A, B and C, entering into Eqs. (S13)-(S15), as follows:

Let $S^{(b)}\left(t_j^{(b)}\right)$ and $\tilde{S}_k^{(b)}\left(t_j^{(b)}\right)$ denote, respectively, the mean detector input signal and the recorded CCD detector output signal for the kth bead in the sample, both at observation time $t_j^{(b)}$.

$$\tilde{S}_k^{(b)}\left(t_j^{(b)}\right) = S^{(b)}\left(t_j^{(b)}\right) + \eta_k^{(b)}\left(t_j^{(b)}\right) \tag{S17}$$

The mean bead signal, $S^{(b)}\left(t_j^{(b)}\right)$, represents the mean fluorescent photon count per bead emitted into the CCD detector at observation time $t_j^{(b)}$, averaged over all possible bead sizes. It therefore does not depend on the bead index k. The bead noise variables, $\eta_k^{(b)}\left(t_j^{(b)}\right)$, contain the random contributions from *both* the detection noise *and* from the bead size variability. They are assumed to be statistically independent across all beads (but not across all times!), with zero mean and a total noise variance $\left(\sigma_{\eta,j}^{(b)}\right)^2$:

$$\left\langle \eta_k^{(b)}(t_j^{(b)}) \;\; \eta_{k'}^{(b)}(t_j^{(b)}) \right\rangle_{e,b} = \left(\sigma_{\eta,j}^{(b)}\right)^2 \delta_{k,k'} \tag{S18}$$

Here, the brackets $\langle \ldots \rangle_{e,b}$ denote the joint population average over all possible experimental detection noise configurations *and* over all possible bead sizes.

Applying the same noise compounding and propagation analysis as for Eq. (S13), but including *both* the same six detection noise sources as in Eq. (S13) *and* in addition the bead size variablility, we can then show that

$$\left(\sigma_{\eta,j}^{(b)}\right)^2 = C^{(b)} + A^{(b)} S^{(b)}\left(t_j^{(b)}\right) + B^{(b)}\left(S^{(b)}\left(t_j^{(b)}\right)\right)^2 \tag{S19}$$

where coefficients A, B and C, in Eq. (S13) are related to $A^{(b)}$, $B^{(b)}$ and $C^{(b)}$, by

$$A = A^{(b)}, \qquad C = C^{(b)}, \qquad B = \frac{B^{(b)} - \sigma_B^2}{1 + \sigma_B^2}\ . \tag{S20}$$

Here, σ_B^2 is the relative variance of the emitted fluorescent photon count per bead, due to bead size variability only, in the absence of any detection noise. This is discussed in more detail below. From the mathematical noise compounding and propagation analysis it follows that the coefficients A, B and C, in Eq. (S13), as well as $A^{(b)}$, $B^{(b)}$ and $C^{(b)}$, in Eq. (S19) must be non-negative. This requirement will allow us to impose important constraints on the bead parameters, as discussed below.

For a sufficiently large bead sample size, $K^{(b)}$, we can then estimate the $\sigma_\eta^{(b)}$-values and corresponding $S^{(b)}$-values from sample means of the observed bead data, $\tilde{S}_k^{(b)}\left(t_j^{(b)}\right)$:

$$S^{(b)}\left(t_j^{(b)}\right) = \frac{1}{K^{(b)}} \sum_{k=1}^{K^{(b)}} \tilde{S}_k^{(b)}\left(t_j^{(b)}\right) \tag{S21}$$

$$\left(\sigma_{\eta,j}^{(b)}\right)^2 = \frac{1}{(K^{(b)}-1)} \sum_{k=1}^{K^{(b)}} \left[\tilde{S}_k^{(b)}\left(t_j^{(b)}\right) - S^{(b)}\left(t_j^{(b)}\right)\right]^2 \tag{S22}$$

where $K^{(b)}$ is the total number of beads observed in our experiment at time $t_j^{(b)}$.

Fig. S7 shows a plot of the combined results for $\left(\sigma_{\eta,j}^{(b)}\right)^2$ *vs.* $S^{(b)}\left(t_j^{(b)}\right)$ from six bead experiments, labeled Experiment 0, 1, 2, 3, 4, and 5 below. Experiment 0 was identical to the cell experiment, in terms of illumination intensity and CCD exposure time, and it ran for 10 days. Experiments 1-5 were only run for 1 day each, with reduced illumination intensity and successively reduced CCD exposure times. The experiment with the highest fluorescent photon counts was thus Experiment 0, and the experiment with the lowest photon counts was Experiment 5. The observed numbers of beads in the six experiments were $K^{(b)} = 546$, 305, 279, 284, 285, 262 for Experiment 0, 1, 2, 3, 4 and 5, respectively. The fluorescent photon counts in the bead experiments bracketed those used in the cell experiments. Also shown in Fig. S7 is the result of a least-squares fit of Eq. (S19), subject to the non-negativity constraint imposed on each fit parameter, $A^{(b)}$, $B^{(b)}$ and $C^{(b)}$. Fig. S8 shows an analogous plot and fit for the data from Experiments 1-5 only. Both fits were remarkably close to the data, both with $R^2 > 0.99$. The fit parameter results are summarized in Table S1. It is important to notice that the $B^{(b)}$-term dominates in Eq. (S19), relative to the $A^{(b)}$- and $C^{(b)}$-terms, over the relevant range of $S^{(b)}\left(t_j^{(b)}\right)$-values, which is of order 10^6, measured in our CCD camera output units.

To estimate the parameter σ_B^2, we need to model how each bead's emitted photon count, S_B, depends on the bead size, quantified in terms of the bead diameter, D. This dependence is controlled by the spatial distribution of the fluorescent dopant inside the bead. As a simplest model of the bead doping profile, we assume that some fraction of the dopant material is uniformly distributed, with a D-independent bulk dopant density, over the 3D spherical volume of the bead, while the remaining fraction of dopant material is uniformly distributed over the bead's 2D spherical surface, with D-independent surface dopant density. The total number of fluorescent photons, S_B, emitted by a bead into the CCD detector, is assumed to be proportional to the total dopant amount in the bead and proportional to the number of incident illumination photons, I, during exposure. Hence, we can write S_B as a function of D the form

$$S_B(D) = I\left[a_{B3}\left(\frac{D}{\bar{D}}\right)^3 + a_{B2}\left(\frac{D}{\bar{D}}\right)^2\right] = \bar{S}_B\left[f_B\left(\frac{D}{\bar{D}}\right)^3 + \left(1-f_B\right)\left(\frac{D}{\bar{D}}\right)^2\right]. \tag{S23}$$

The first and second term in Eq. (S20) represent, respectively, the volume and surface dopant contributions to the total fluorescence: they are proportional to the bead volume, $(4\pi/3)(D/2)^3$, and to the bead surface area, $4\pi(D/2)^2$, respectively, rescaled by the

mean bead diameter, $\bar{D}$. The coefficients a_{B3} and a_{B2} are proportional to the dopant bulk and dopant surface density, respectively, and hence assumed to be independent of D. The first square bracket term in (S20) is thus proportional to the bead's total dopant content. Rewriting (S23) in terms of $\bar{S}_B = I\left(a_{B3}+a_{B2}\right)$ and $f_B = a_{B3}/\left(a_{B3}+a_{B2}\right)$ we get:

$$0 \leq f_B \leq 1 \ . \tag{S24}$$

The parameter σ_B^2, the relative variance of $S_B(D)$, is then given by

$$\sigma_B^2 = \left\langle \left[S_B(D) - \left\langle S_B(D) \right\rangle_b \right]^2 \right\rangle_b \Big/ \left\langle S_B(D) \right\rangle_b^2 . \tag{S25}$$

where $\langle \ldots \rangle_b$ denotes the population mean over the bead sizes D. Since the beads in our experiments had a mean diameter of $\bar{D} \equiv \langle D \rangle_b = 9.94\mu\text{m}$ with a standard deviation of only $\sigma_D \equiv \left\langle \left(D - \langle D \rangle_b \right)^2 \right\rangle_b^{1/2} = 0.76\mu\text{m}$, we expand $S_B(D)$ in Eq. (S22) around $\bar{D}$ to linear order in $(D-\bar{D})$, to obtain

$$\sigma_B^2 = (2+f_B)^2 \left(\frac{\sigma_D}{\bar{D}} \right)^2 \ . \tag{S26}$$

From Eq. (S24) it then follows that σ_B^2 is limited to the range of values

$$\sigma_{B,\text{Lo}}^2 \leq \ \sigma_B^2 \leq \ \sigma_{B,\text{Hi}}^2 \tag{S27}$$

where

$$\sigma_{B,\text{Lo}}^2 = \ 4\left(\frac{\sigma_D}{\bar{D}} \right)^2 \cong \ 0.023383 \qquad \text{and} \qquad \sigma_{B,\text{Hi}}^2 = \ 9\left(\frac{\sigma_D}{\bar{D}} \right)^2 \cong \ 0.052613 \ . \tag{S28}$$

The values of f_B or σ_B^2 can not be extracted directly from available experimental data. However, Eqs. (S24) and, respectively, Eqs. (S27) and (S28) do already constrain their allowed ranges. Furthermore, when combined with Eqs. (S20) and (S26), the non-negativity constraint on the B - coefficient,

$$B \geq 0 \ , \tag{S29}$$

imposes an additional upper bound on f_B and σ_B^2 so that

$$0 \leq f_B \leq f_{B,\max} \equiv \min\left[1, \ \left(\left(\frac{\bar{D}}{\sigma_D} \right)^2 B^{(b)} - 2 \right) \right] \ , \quad \sigma_{B,\text{Lo}}^2 \leq \sigma_B^2 \leq \sigma_{B,\max}^2 \equiv \min\left(\sigma_{B,\text{Hi}}^2, B^{(b)} \right) \tag{S30}$$

Resulting $f_{B,\max}$- and $\sigma_{B,\max}^2$-values are shown in Table S1 for the two $B^{(b)}$-values extracted from the fits of Eq. (S19) to the Experiment 1-5 and Experiment 0-5 bead data sets. As discussed below, other non-negativity constraints will also impose very tight lower bounds, $f_{B,\min}$ and $\sigma_{B,\min}^2$, which then limits f_B and σ_B^2 to an even narrower range of allowed values, as also shown in Table S1. We therefore adopt the "volume fraction" variable, f_B, as a convenient model input parameter to quantify the effect of the bead

doping profile on the variability of the bead fluorescent photon output, *via* Eqs. (S26) and (S20).

- De-Trending of RB-Normalized Time Series Data

A first step in any periodogram analysis is to ensure that the underlying time series of fluorescence for each cell is stationary. Because of photobleaching (Fig. 2B), the time series of *ccg-2* fluorescence for each cell was not stationary. It was therefore necessary to carry out a moving average de-trending[8] on the RB-normalized cell time series data, $\tilde{Y}_k(t_j)$, from Eq. (S11). The first step in this procedure was to subtract from the data a linear trend line, $y_k^{(m)}(t) \equiv \alpha_k^{(m)} t + \beta_k^{(m)}$, fitted to the data over short contiguous time blocks, each block containing $M = 48$ successive time points, t_j, as follows:

$$\begin{aligned} \tilde{V}_k^{(m)}(t_j) &= \tilde{Y}_k(t_j) - \alpha_k^{(m)} t_j - \beta_k^{(m)} \quad \text{if } j = m, \ldots, j_m \equiv m + M - 1 \\ &\equiv 0 \quad \text{otherwise.} \end{aligned} \tag{S31}$$

Here, the block index, m, runs over the values $m = 1, 2, \ldots, L - M + 1$. The regression coefficients, $\alpha_k^{(m)}$ and $\beta_k^{(m)}$, in this moving average de-trending were estimated by linear least-squares fit to the data points $\tilde{Y}_k(t_j)$ from the respective time block, $j = m, \ldots, j_m$:

$$\alpha_k^{(m)} = c_m \sum_{j=m}^{j_m} \sum_{j'=m}^{j_m} \left(M\delta_{j,j'} - 1 \right) t_{j'} \tilde{Y}_k(t_j) \tag{S32}$$

and

$$\beta_k^{(m)} = \frac{1}{M} \sum_{j=m}^{j_m} \left[\tilde{Y}_k(t_j) - \alpha_k^{(m)} t_j \right] \tag{S33}$$

with

$$c_m = \left[M \sum_{j=m}^{j_m} t_j^2 - \left(\sum_{j=m}^{j_m} t_j \right)^2 \right]^{-1}. \tag{S34}$$

The final de-trended signal, denoted by $\tilde{V}_k(t_j)$, was then obtained by averaging the block-de-trended data, $\tilde{V}_k^{(m)}(t_j)$, from all those time blocks which contain the time point t_j, *i.e.*, over all m-values for which $m \le j \le j_m$. Combining that averaging procedure with Eqs. (S28), (S29) and (S30), the resulting $\tilde{V}_k(t_j)$ can be written as a linear transformation of the non-de-trended RB-normalized CCD output data, $\tilde{Y}_k(t_j)$, with a transformation "weight" matrix $w(j, j')$ as follows:

$$\tilde{V}_k(t_j) = \sum_{j'=1}^{L} w(j, j')\, \tilde{Y}_k(t_{j'}) \tag{S35}$$

where

$$w(j,j') = b_j^{-1} \sum_{m=1}^{L-M+1} w_m(j,j') \tag{S36}$$

and

$$\begin{aligned} w_m(j,j') &= h_m(j)\ \delta_{j,j'} \\ &\quad - h_m(j) h_m(j') \sum_{j''=1}^{L} h_m(j'') \left[c_m \left(t_j - \bar{t}_m \right) t_{j''} \left(M \delta_{j',j''} - 1 \right) + \frac{\delta_{j',j''}}{M} \right] . \end{aligned} \tag{S37}$$

The m th block indicator function, $h_m(j)$, is given by

$$h_m(j) = 1 \quad \text{if } m \le j \le j_m, \quad \text{else} \quad h_m(j) = 0 , \tag{S38}$$

and the mean block time $\bar{t}_m$ is

$$\bar{t}_m \equiv \frac{1}{M} \sum_{j=m}^{j_m} t_j \ . \tag{S39}$$

The quantity b_j is the number of time blocks, m , which contain observation time t_j :

$$b_j = \sum_{m=1}^{L-M+1} h_m(j) \ . \tag{S40}$$

By Eq. (S11) we then get, for cell # k at observation time t_j :

$$\tilde{V}_k\ (t_j) = V_k\ (t_j) + \zeta_k(t_j) \ . \tag{S41}$$

Here, $\zeta_k(t_j)$ is the de-trended, RB-normalized experimental detection noise and $V_k\ (t_j)$ is the de-trended, RB-normalized fluorescent signal in the absence of experimental detection noise:

$$V_k\ (t_j) = \sum_{j'=1}^{L} w(j,j')\ Y_k\ (t_{j'}) \tag{S42}$$

$$\zeta_k(t_j) = \sum_{j'=1}^{L} w(j,j')\ \varepsilon_k(t_{j'}) \tag{S43}$$

The noise variables $\zeta_k(t_j)$ are not statistically independent anymore at different times and their (co-) variance matrix elements, obtained *via* Eqs. (S43) and (S12), are

$$\left\langle\ \zeta_k(t_j)\ \zeta_{k'}(t_{j'})\ \right\rangle_e = \sigma_\varepsilon^2 \delta_{k,k'} \gamma(j,j') \tag{S44}$$

where

$$\gamma(j,j') = \sum_{j''=1}^{L} w(j,j'')\ w(j',j'')\ \rho_{j''}^2 \tag{S45}$$

$$\rho_j^2 = \sigma_{\varepsilon,j}^2 / \sigma_\varepsilon^2 \tag{S46}$$

$$\sigma_\varepsilon^2 = \frac{1}{L} \sum_{j=1}^{L} \sigma_{\varepsilon,j}^2 \tag{S47}$$

$$\sigma_{\varepsilon,j}^2 = \frac{\sigma_{\xi,j}^2}{(Z(t_j))^2} \tag{S48}$$

The variances $(\sigma_{\xi,j})^2$ in Eq. (S48) were estimated from the observed CCD data by Eq. (S15) and (S16), with A-, B- and C-coefficients estimated from the bead experiments by Eq. (S20).

- <u>Observed Cell-Averaged Periodogram and Periodogram Variances</u>

The cell-averaged periodogram in Eq.(4), $Q^{\text{obs}}(f_\ell)$, that can be directly compared to the model prediction, $Q^{\text{exp}}(f_\ell)$, must be defined in terms of the de-trended, RB-normalized signals, $V_k(t_j)$, that would be observed in the *absence* of detection noise, as follows:

$$Q^{\text{obs}}(f_\ell) = \langle Q(f_\ell) \rangle_c = \langle Q_k(f_\ell) \rangle_c \quad . \tag{S49}$$

Here, $\langle \dots \rangle_c$ again denotes the population mean over the intra-cellular stochastic time series, $X_k(t_j)$ and

$$Q(f_\ell) \equiv \frac{1}{K} \sum_{k=1}^{K} Q_k(f_\ell) \tag{S50}$$

and

$$Q_k(f_\ell) \equiv \left| F_k(f_\ell) \right|^2 \tag{S51}$$

is the periodogram of the fluorescent signal $V_k(t_j)$ and $F_k(f_\ell)$ is its Fourier transform, at frequencies $f_\ell = \ell / T$ with $\ell = 0,1,\dots,L$ and imaginary unit *i* with $i^2 \equiv -1$:

$$F_k(f_\ell) = \frac{1}{L} \sum_{j=1}^{L} \exp(-2\pi i \, f_\ell t_j) \; V_k(t_j) \; . \tag{S52}$$

Note that, in Eq. (S49), the population mean, $\langle Q_k(f_\ell) \rangle_c$, taken over all intra-cellular stochastic time series, $X_k(t_j)$, is again independent of the cell index *k*, analogous to Eq. (S14).

The de-trended signal, $V_k(t_j)$, in Eq. (S52) is based, by linear transformation, on the "clean" fluorescent signal, $Y_k(t_j)$, without experimental detection noise. Therefore, $Q^{\text{obs}}(f_\ell)$, cannot be directly calculated from its defining Eqs. (S49)-(S52). Instead, we must express $Q^{\text{obs}}(f_\ell)$ in terms of the actually observed, RB-normalized CCD signal, $\tilde{Y}_k(t_j)$, which *is* subject to experimental detection noise. The result is as follows:

$$Q^{\text{obs}}(f_\ell) \; = \; \tilde{Q}^{\text{obs}}(f_\ell) \; - \; Q^{\text{bias}}(f_\ell) \quad , \tag{S53}$$

$$\tilde{Q}^{\text{obs}}(f_\ell) \; \equiv \; \langle \tilde{Q}(f_\ell) \rangle_{e,c} \; = \; \langle \tilde{Q}_k(f_\ell) \rangle_{e,c} \quad . \tag{S54}$$

Here, $\langle ... \rangle_{e,c}$ again denotes the joint population mean over both the experimental detection noise and over the intra-cellular stochastic time series, $X_k(t_j)$, and

$$\tilde{Q}(f_\ell) \equiv \frac{1}{K}\sum_{k=1}^{K}\tilde{Q}_k(f_\ell) \quad , \tag{S55}$$

$$\tilde{Q}_k(f_\ell) \equiv \left|\tilde{F}_k(f_\ell)\right|^2 \quad , \tag{S56}$$

$$\tilde{F}_k(f_\ell) \equiv \frac{1}{L}\sum_{j=1}^{L} \exp(-2\pi i\ f_\ell\, t_j)\ \tilde{V}_k(t_j) \ , \tag{S57}$$

with $\tilde{V}_k(t_j)$ calculated by Eq. (S11) and Eqs. (S35)-(S40), from the observed CCD output signal $\tilde{S}_k(t_j)$. Again, $\left\langle \tilde{Q}_k(f_\ell) \right\rangle_{e,c}$ in Eq. (S54) does not depend on the cell index *k*. The bias subtraction correction in Eq. (S53), caused by the experimental detection noise, is given by

$$Q^{\text{bias}}(f_\ell) \ = \ \frac{\sigma_\varepsilon^2}{L}\gamma_Q(\ell) \quad , \tag{S58}$$

$$\gamma_Q(\ell) \ \equiv \ L\sum_{j=1}^{L}\left|w_Q(\ell,j)\right|^2 \rho_j^2 \quad , \tag{S59}$$

$$w_Q(\ell,j) \ \equiv \ \frac{1}{L}\sum_{j'=1}^{L} \exp(-2\pi i\ f_\ell\, t_{j'})\ w(j',j) \quad , \tag{S60}$$

where, σ_ε^2, ρ_j^2 and $w(j',j)$ were calculated by Eq. (S47), Eq. (S46) and Eqs. (S37), (S36), respectively. For a large cell sample size, *K*, we approximate the population mean in Eq. (S54) by the sample mean, *i.e.*,

$$\tilde{Q}^{\text{obs}}(f_\ell) \ \cong \ \tilde{Q}(f_\ell) \quad . \tag{S61}$$

We thus used Eq. (S53), combined with Eqs. (S55)-(S61), to calculate $Q^{\text{obs}}(f_\ell)$.

The total variance of the periodogram $\tilde{Q}^{\text{obs}}(f_\ell)$, denoted by σ_ℓ^2 ; the contribution to this variance from the stochastic intra-cellular variability, denoted by $(\sigma_\ell^c)^2$; and the contribution from the experimental detection noise, denoted by $(\sigma_\ell^e)^2$, are defined by:

$$\sigma_\ell^2 \equiv \left\langle\left[\tilde{Q}(f_\ell)-\left\langle\tilde{Q}(f_\ell)\right\rangle_{e,c}\right]^2\right\rangle_{e,c} = \left\langle\left(\tilde{Q}(f_\ell)\right)^2\right\rangle_{e,c} - \left\langle\tilde{Q}(f_\ell)\right\rangle_{e,c}^2 \ , \tag{S62}$$

$$(\sigma_\ell^c)^2 \equiv \left\langle\left[Q(f_\ell)-\left\langle Q(f_\ell)\right\rangle_c\right]^2\right\rangle_c \quad , \tag{S63}$$

$$(\sigma_\ell^e)^2 \equiv \ \sigma_\ell^2 - \ (\sigma_\ell^c)^2 \tag{S64}$$

where $Q(f_\ell)$ and $\tilde{Q}(f_\ell)$ are the sample averages given by Eqs. (S50) and (S55).

To estimate σ_ℓ^2, we bootstrapped our sample of K=1591 single-cell trajectories, $\tilde{V}_k(t_j)$, to generate a sample of 100,000 bootstrap estimates for $\tilde{Q}(f_\ell)$. The population means $\left\langle \tilde{Q}(f_\ell) \right\rangle_{e,c}$ and $\left\langle \left(\tilde{Q}(f_\ell) \right)^2 \right\rangle_{e,c}$ in Eq. (S62) were then approximated by the respective sample means over the bootstrap sample.

We then evaluated $(\sigma_\ell^c)^2$ and $(\sigma_\ell^e)^2$ from the following relations, derived from Eqs. (S62)-(S64):

$$(\sigma_\ell^e)^2 = \frac{2\sigma_\varepsilon^2}{KL} \left[\left\langle \tilde{Q}(f_\ell) \right\rangle_{e,c} \gamma_Q(\ell) + \mathrm{Re}\left(\left\langle \tilde{R}(f_\ell) \right\rangle_{e,c} \beta_Q(\ell)^* \right) \right] - \frac{\sigma_\varepsilon^4}{KL^2} \left[\left| \gamma_Q(\ell) \right|^2 + \left| \beta_Q(\ell) \right|^2 \right] , \tag{S65}$$

$$(\sigma_\ell^c)^2 = \sigma_\ell^2 - (\sigma_\ell^e)^2 \quad . \tag{S66}$$

where

$$\tilde{R}(f_\ell) \equiv \frac{1}{K} \sum_{k=1}^{K} \left(\tilde{F}_k(f_\ell) \right)^2 \quad , \tag{S67}$$

$$\beta_Q(\ell) \equiv L \sum_{j=1}^{L} \left(w_Q(\ell, j) \right)^2 \rho_j^2 \quad . \tag{S68}$$

In Eq. (S65), $\beta_Q(\ell)^*$ denotes the complex conjugate of $\beta_Q(\ell)$. The quantities $\gamma_Q(\ell)$, σ_ε^2, $w_Q(\ell, j)$ and ρ_j^2 were obtained from Eqs. (S59), (S47), (S60) and (S46), respectively. The population average $\left\langle \tilde{R}(f_\ell) \right\rangle_{e,c}$ in Eq. (S65) was approximated by the sample average over the same bootstrap sample that was used to estimate σ_ℓ^2.

By definition, the above-defined quantities $Q^{\mathrm{obs}}(f_\ell)$, $(\sigma_\ell^e)^2$ and $(\sigma_\ell^c)^2$ must be non-negative. When combined with the results for $Q^{\mathrm{obs}}(f_\ell)$ in Eq.(53), for $(\sigma_\ell^e)^2$ in Eq. (S65) and for $(\sigma_\ell^c)^2$ in Eq. (S66), each of these three non-negativity constraints implies a respective upper bound on the detection noise variance parameter σ_ε^2. The values of $Q^{\mathrm{obs}}(f_\ell)$, $(\sigma_\ell^e)^2$ and $(\sigma_\ell^c)^2$ are each becoming negative when σ_ε^2 becomes too large. By Eq. (S53), (S65) and (S66), we get upper bounds on σ_ε^2, denoted by $\sigma_{\varepsilon,\max 1}^2$, $\sigma_{\varepsilon,\max 2}^2$, $\sigma_{\varepsilon,\max 3}^2$, respectively, as follows:

$$\sigma_{\varepsilon,\max 1}^2 = \min_{\ell=0,\ldots,(L-1)} \left(\tilde{Q}^{\mathrm{obs}}(f_\ell) \, \frac{L}{\gamma_Q(\ell)} \right) \tag{S69}$$

$$\sigma_{\varepsilon,\max 2}^2 = \min_{\ell=0,\ldots,(L-1)} \frac{2L \left(\left\langle \tilde{Q}(f_\ell) \right\rangle_{e,c} \gamma_Q(\ell) + \mathrm{Re}\left(\left\langle \tilde{R}(f_\ell) \right\rangle_{e,c} \beta_Q(\ell)^* \right) \right)}{\left| \gamma_Q(\ell) \right|^2 + \left| \beta_Q(\ell) \right|^2} \tag{S70}$$

$$\sigma^2_{\varepsilon,\max3} = \min_{\ell=0,\ldots,(L-1)} \frac{KL\,\sigma^2_\ell}{2\left(\left\langle \tilde{Q}(f_\ell)\right\rangle_{e,c} \gamma_Q(\ell) + \mathrm{Re}\left(\left\langle \tilde{R}(f_\ell)\right\rangle_{e,c} \beta_Q(\ell)^*\right)\right)} \quad \text{(S71)}$$

Here, Eq. (S71) holds under the condition that $\sigma^2_\varepsilon << \sigma^2_{\varepsilon,\max2}$, so that the σ^4_ε-term in Eq. (S65) is negligible compared to the σ^2_ε-term. From our ~~experimental~~ data, we find that $\sigma^2_{\varepsilon,\max1}$ is the most restrictive, *i.e.*, the lowest upper bound on σ^2_ε, and hence, it is the overall upper bound:

$$\sigma^2_{\varepsilon,\max} \equiv \min\left(\sigma^2_{\varepsilon,\max1}, \sigma^2_{\varepsilon,\max2}, \sigma^2_{\varepsilon,\max3}\right) = \min_{\ell=0,\ldots,(L-1)} \left(\tilde{Q}^{\mathrm{obs}}(f_\ell)\, \frac{L}{\gamma_Q(\ell)}\right) \quad \text{(S72)}$$

By Eqs. (S47), (S48) and (S15), this upper bound imposed on σ^2_ε also implies an upper bound on the B-coefficient entering into Eq. (S15), since $\sigma^2_{\xi,j}$ and hence σ^2_ε are monotonically increasing functions of B, given the magnitudes of A, B, and C of interest in our data analysis, as shown in Table S1. By Eqs. (S20) and (S26), the *upper* bounds imposed on σ^2_ε thus imply corresponding *lower* bounds imposed on the unknown parameter values σ^2_B and f_B. These upper and, respectively, lower bounds are also shown in Table S1, denoted by $B_{\max}$, $\sigma^2_{B,\min}$ and $f_{B,\min}$. As can be seen from Table S1, these upper/lower bounds on B, σ^2_B and f_B, combined with the bounds, $B_{\min} \equiv 0$, $\sigma^2_{B,\max}$ and $f_{B,\max}$, constrain B, σ^2_B and f_B to very narrow allowed ranges.

Fig. S9 shows the results for the bias-corrected periodogram, $Q^{\mathrm{obs}}(f_\ell)$, plotted *vs.* the frequency f_l for a sequence of increasing f_B-values. The $(\sigma_{\xi,j})^2$-values entering into $Q^{\mathrm{obs}}(f_\ell)$-results were calculated by Eq. (S15), based on the bead noise calibration coefficients, $A^{(b)}$, $B^{(b)}$ and $C^{(b)}$, from the bead Experiments 1-5, see Fig. S8 and Table S1. Notice that $Q^{\mathrm{obs}}(f_\ell)$-values become negative as f_B is lowered below $f_{B,\min}$. However, initially, for f_B-values below, but still close to $f_{B,\min}$, this happens only at the shortest periods ($T_\ell \equiv 1/f_\ell$). As f_B is lowered further, the range of negative-$Q^{\mathrm{obs}}(f_\ell)$ periods with increases.

The top two $Q^{\mathrm{obs}}(f_\ell)$-curves in Fig. S9 show what happens when f_B is varied over the allowed range: from $f_{B,\min} \cong 0.7679$ to $f_{B,\max} \cong 0.7775$, $Q^{\mathrm{obs}}(f_\ell)$ varies by no more than 10% of its peak value, $\max_\ell\left[Q^{\mathrm{obs}}(f_\ell)\right] \approx 0.04$, across all periods $T_\ell \equiv 1/f_\ell$. Clearly, $Q^{\mathrm{obs}}(f_\ell)$ is not substantially affected by the choice of f_B-value within the allowed range $f_{B,\min} \leq f_B \leq f_{B,\max}$.

As an alternative approach to estimating σ_B^2 and f_B we also exploited the time dependence of the relative bead noise correlations, defined by

$$s^{(b)}(j,j') = \frac{\left\langle \eta_k^{(b)}(t_j^{(b)})\ \eta_k^{(b)}(t_{j'}^{(b)}) \right\rangle_{e,b}}{\left\langle S_k^{(b)}(t_j^{(b)}) \right\rangle_{e,b} \left\langle S_k^{(b)}(t_{j'}^{(b)}) \right\rangle_{e,b}} . \tag{S73}$$

Thus, $s^{(b)}(j,j')$ is the temporal correlation between the noise variables $\eta_k^{(b)}(t_j^{(b)})$ and $\eta_{k'}^{(b)}(t_{j'}^{(b)})$, normalized by their respective mean signals, $\left\langle S_k^{(b)}(t_j^{(b)}) \right\rangle_{e,b}$ and $\left\langle S_{k'}^{(b)}(t_{j'}^{(b)}) \right\rangle_{e,b}$, on the same bead, $k = k'$, at bead observation times $t_j^{(b)}$ and $t_{j'}^{(b)}$. [Note here that $s^{(b)}(j,j) = \left(\sigma_{\eta,j}\right)^2 \Big/ \left\langle S_k^{(b)}(t_j^{(b)}) \right\rangle_{e,b}^2$ for $t_j^{(b)} = t_{j'}^{(b)}$.] By averaging over all beads in the six bead data sets, Experiments 0-5 and replacing population averages $\left\langle \ldots \right\rangle_{e,b}$ in Eq. (S73) by sample averages analogous to Eqs. (S21) and (S22), we are able to extract reasonable estimates for $s^{(b)}(j,j')$.

The basic idea of the σ_B^2-estimation from time correlations then rests on the observation that, on physical grounds, the correlation contribution to $s^{(b)}(j,j')$ from all the six detection noise sources will die out as a function of temporal distance,

$$\Delta t(j,j') \equiv \left| t_j^{(b)} - t_{j'}^{(b)} \right| , \tag{S74}$$

since the detection noise sources are subject to random temporal fluctuations. On the other hand, the "noise" contribution from the bead size variation does not die out, since the bead size, D, does not fluctuate with time. As a simple physical model, we assume that the detection noise correlations die out exponentially with $\Delta t(j,j')$, *i.e.*, with a functional dependence of the form

$$s^{(b)}(j,j') = a_s \exp\left(-\frac{\Delta t(j,j')}{\tau_s} \right) + b_s \quad . \tag{S75}$$

The constants a_s, τ_s and b_s denote, respectively, the equal-time ($t_j^{(b)} = t_{j'}^{(b)}$) contribution from the temporally fluctuating detection noise sources, the exponential decay lifetime of the temporally fluctuating detection noise, and the contribution from the temporally constant bead size variation. In the limit $\Delta t(j,j') \rightarrow \infty$, only the bead size variance contribution to $s^{(b)}(j,j')$ survives. Recall now that, σ_B^2 is the relative variance of the emitted fluorescent photon count per bead, due to bead size variability only, in the absence of detection noise. The model constant b_s is then directly related to the relative bead variance parameter, σ_B^2, by

$$\sigma_B^2 = \lim_{\Delta t(j.j') \rightarrow \infty} s^{(b)}(j,j') = b_s . \tag{S76}$$

We therefore extracted $s^{(b)}(j,j')$ from the six bead data sets obtained in Experiments 0-5. We then fitted those $s^{(b)}(j,j')$-data with a fitting function of the form given by Eq. (S75),

with fit parameters, a_s, τ_s and b_s. The resulting values for σ_B^2 from Eq. (S76), were $\sigma_B^2 = 0.04778 \pm 9.3\times10^{-5}$ if all six Experiment 0-5 data were included, and $\sigma_B^2 = 0.03864 \pm 1.1\times10^{-4}$ if only the five Experiment 1-5 data were included,. The given standard deviations of σ_B^2 were estimated by bootstrapping the respective bead data sets over all beads, $k = 1,...,K^{(b)}$, for each of the six or five bead experiments, and then performing a separate fit on every bootstrap data set thus generated. As seen in Table S1, the resulting two estimates bracketed the desired range $\sigma_{B,\min}^2 \le \sigma_B^2 \le \sigma_{B,\max}^2$ and they were within 7% of that range. We conclude that the basic model assumptions underlying our detection and bead size noise analysis is well supported.

- Ensemble Fitting of the Stochastic Model by Markov Chain Monte Carlo

The standard Markov chain Monte Carlo (MC) ensemble simulation method[9], based on the $\chi^2(\Theta)$ fitting criterion from Eq.(4), was adapted to find and sample the model parameter space in Θ-regions of near-minimal $\chi^2(\Theta)$. The standard ensemble probability distribution (likelihood)[9],

$$L(\Theta) = \frac{1}{\Omega}\exp\left(-\frac{1}{2}\chi^2(\Theta)\right) , \tag{S77}$$

was used, with Ω denoting the normalization factor. The objective of the ensemble simulation is then to generate a large random sample of parameter variable vectors, Θ, drawn from $L(\Theta)$, which provide near-optimal fits to the experimental data.

In principle, we could have constructed $\chi^2(\Theta)$ from the squared residuals of the bias-corrected bare (un-normalized) observed periodogram, $Q^{\text{obs}}(f_\ell)$ from Eq. (S53), and the corresponding bare expected periodogram, $Q^{\exp}(f_\ell;\Theta)$ from Eq. (S4). However, in light of the relative smallness of the above-estimated detection noise contributions to the variance and bias of the observed bare periodogram, we employed a simplified approach which neglects these small detection noise corrections. This simplified approach also eliminates the uncertainties due to the unknown unit conversion factor variable ϕ entering into the model predictions *via* Eq. (S5). This is achieved by defining $\chi^2(\Theta)$ in terms *normalized* instead of *bare* periodograms, as was done in Eq. (4). The normalized observed periodogram in Eq. (4) is defined by

$$\bar{Q}^{\text{obs}}(f_\ell) = \frac{1}{K}\sum_{k=1}^{K}\frac{1}{\tilde{P}_k}\tilde{Q}_k(f_\ell) \tag{S78}$$

where $\tilde{Q}_k(f_\ell)$ is given by Eqs.(S56) and (S57) and the normalization factors are

$$\tilde{P}_k = \sum_{\ell=1}^{[L/2]} \tilde{Q}_k(f_\ell) \qquad \text{for} \quad k = 1,...,K \, . \tag{S79}$$

Likewise, the normalized expected periodogram in Eq. (4) is defined by

$$\bar{Q}^{\exp}(f_\ell;\Theta) = \frac{1}{K^{(\text{Model})}} \sum_{k=1}^{K^{(\text{Model})}} \frac{1}{P_k^{\exp}(\Theta)} Q_k^{\exp}(f_\ell;\Theta) \tag{S80}$$

where $Q_k^{\exp}(f_\ell;\Theta)$ is given by Eq. (S5) and the normalization factors are

$$P_k^{\exp}(\Theta) = \sum_{\ell=1}^{[L/2]} Q_k^{\exp}(f_\ell;\Theta) \qquad \text{for} \quad k = 1,...,K^{(\text{Model})} \, . \tag{S81}$$

Lastly, the experimental variances in Eq. (4) are given by

$$\left(\bar{\sigma}_\ell\right)^2 = \frac{1}{K-1} \sum_{k=1}^{K} \left[\frac{1}{P_k} \tilde{Q}_k(f_\ell) - \bar{Q}^{\text{obs}}(f_\ell) \right]^2 \, . \tag{S82}$$

Since $Q_k^{\exp}(f_\ell;\Theta)$ and $P_k^{\exp}(\Theta)$, by Eqs. (S5) and (S81), are both proportional to the unit conversion factor variable, ϕ, this factor cancels out in Eq. (S80). Hence, neither $\bar{Q}^{\exp}(f_\ell;\Theta)$ nor $\chi^2(\Theta)$ depends on ϕ. Due to the normalization factors in Eqs. (S78) and (S80), both $\ell = 1,...,[L/2]$ and $\bar{Q}^{\exp}(f_\ell;\Theta)$ are dimensionless and normalized to unity, *i.e.*,

$$\sum_{\ell=1}^{[L/2]} \bar{Q}^{\text{obs}}(f_\ell) = \sum_{\ell=1}^{[L/2]} \bar{Q}^{\exp}(f_\ell;\Theta) = 1 \, . \tag{S83}$$

The $\bar{Q}^{\text{obs}}(f_\ell)$ and $\bar{Q}^{\exp}(f_\ell;\Theta)$ can therefore be compared directly, without any unit conversions, as was done in Eq.(4).

Standard Metropolis MC updating steps, based on $L(\Theta)$, were performed on randomly selected single components of the parameter variable vector,

$$, \qquad \Theta \equiv \left\{ X_1(0), X_2(0), ... X_{N_S}(0), k_1, k_2, ... k_{N_R}, \phi \right\} \tag{S84}$$

except for the ϕ-variable which does not enter into $\chi^2(\Theta)$, as discussed above. Each Metropolis updating step requires a new χ^2-evaluation, for the proposed new Θ-vector. This, in turn, requires the execution of a complete new Gillespie simulation to evaluate the expected normalized model periodogram, $\bar{Q}^{\exp}(f_\ell;\Theta)$, for the proposed new Θ-vector, by Eqs. (S5) and (S80). In each such Gillespie simulation, a total of $K^{(\text{Model})}$ =1024 stochastic trajectories, $X_{n_{\text{obs}},k}(t_j;\Theta)$, were generated, each over a physical simulation time interval of duration $T = 85\text{h}$.

To calculate the observed normalized periodogram, $\bar{Q}^{\text{obs}}(f_\ell)$, entering into Eq.(4), the experimental single-cell fluorescent data, $\tilde{S}_k(t_j)$, and corresponding RB normalization signals, $Z(t_j)$, were taken over the $T = 85\text{h}$ observation time interval starting ***30*** hours and ending at 115 hours to allow stable oscillations and no viability effects at end of the observation time. Using the $w(j, j')$-matrix from Eqs (S34) and (S36)-(S40) the experimental single-cell input data, $\tilde{S}_k(t_j)$, and the RB reference signals, $Z(t_j)$, the de-trended RB-normalized signal, $\tilde{V}_k(t_j)$, was obtained from Eqs. (S11) and (S35). From that, $\bar{Q}^{\text{obs}}(f_\ell)$ was obtained by Eqs. (S56), (S57) and (S80).

At the start of the simulation, the $X_n(0)$- and k_m-variables in Θ were initialized by appropriately rescaled initial concentration and rate coefficient values, respectively, obtained from earlier ensemble simulations of the corresponding deterministic model.[10] (Table S2). A total of 160,000 Metropolis-Hastings updates were performed. The final minimum $\chi^2(\Theta)$ was 130.231, starting at 680.014. A total of 4,706 (=160,000/34) equilibration MC sweeps were then performed where one sweep consists of 34 single-variable Metropolis updating steps as described above. A second equilibration run was tried for an additional 94 sweeps with a resultant $\chi^2(\Theta)$ of ~107 using the model with $\chi^2(\Theta)$ of 130.231 as the initialization. During the accumulation phase, the $\chi^2(\Theta)$ had settled to near-minimal values of about 93.49972 ± 0.366524 with a minimal $\chi^2(\Theta)$ of 84.758. This accumulation phase consisted of a total of 200 accumulation MC sweeps, to collect the random MC sample of Θ-vectors, with one Θ being collected at the end of each MC sweep. What is shown in **Fig. 2D** is the fit corresponding to the minimal $\chi^2(\Theta)$ = 130.231.

Table S1.
Calibration of detector noise obtained from bead experiments (Expts) 0-5 and 1-5. Fit parameter values $C^{(b)}_{\mathrm{fit}}$ $A^{(b)}_{\mathrm{fit}}$ and $B^{(b)}_{\mathrm{fit}}$ are the best fit of Eq. (S19), $(\sigma_{\eta,j}^{(b)})^2 = C^{(b)} + A^{(b)}S^{(b)}(t_j^{(b)}) + B^{(b)}(S^{(b)}(t_j^{(b)}))^2$, subject to the three positivity constraints $C^{(b)} \geq 0$, $A^{(b)} \geq 0$ and $B^{(b)} \geq 0$. The three positivity constraints effectively introduce non-linearity onto the least-squares fitting procedure and can result in best fit parameter choices where one or two of the three fit parameters are forced to assume values of zero exactly, as found in all three fits reported here. The fitted input data, $S^{(b)}(t_j^{(b)})$ and $(\sigma_{\eta,j}^{(b)})^2$, were estimated by Eqs. (S21) and (S22) from the bead fluorescence intensities observed in Expts 0-5. Standard deviations (Std.Dev.) were computed by bootstrapping the bead experiments and repeating the fit for each bootstrap sample. From $B^{(b)}_{\mathrm{fit}}$, we estimated $(\sigma_{B,,\max})^2$ by Eq. (S30). From Eq. (S72), we estimated $(\sigma_{\varepsilon,,\max})^2$, the upper limit on σ_{ε}^2 , which was then used to impose the upper limit on the B-coefficient in Eq.(S15), denoted by $B_{\max}$, *via* Eqs. (S15), (S48) and (S47). Eq. (S20) and $B_{\max}$ were then used to impose the lower σ_B^2-limit, denoted by $(\sigma_{B,\min})^2$. The values of $f_{B,\max}$ and $f_{B,\min}$ were then calculated from $(\sigma_{B,,\max})^2$ and $(\sigma_{B,\min})^2$, respectively, by Eq. (S26). The best fit [1], to the six-experiment bead data set, Expts 0-5, resulted in an empty allowed σ_B^2-range , *i.e.*, $(\sigma_{B,\min})^2 > (\sigma_{B,\max})^2$ or, equivalently, $f_{B,\min} > f_{B,\max}$. The $(\sigma_{B,\min})^2$ and $f_{B,\min}$ from fit [1] also exceeded their physical upper limits, $(\sigma^2_{B,\mathrm{Hi}})^2$ □0.052613 and 1, respectively, imposed by Eq. (S27) and (S24). An additional, more restricted fit [2] to the six-experiment bead data set, Expts 0-5, was then performed, with $A^{(b)}$ forced to $A^{(b)}=0$, and resulted in a finite allowed σ_B^2-range , *i.e.*, $(\sigma_{B,\min})^2 < (\sigma_{B,\max})^2$. The best fit [3], to the five-experiment bead data set, Expt 0-5, resulted in a finite allowed σ_B^2-interval , *i.e.*, $(\sigma_{B,\min})^2 < (\sigma_{B,\max})^2$, without additional restrictions on the fit parameters beyond the three positivity constraints stated above. The results from best fit [3] were used for the subsequent detection error and bias analysis of the single-cell periodogram data.

	Expts	$C^{(b)}_{\mathrm{fit}}$	$A^{(b)}_{\mathrm{fit}}$	$B^{(b)}_{\mathrm{fit}}$	$f_{B,\max}$	$(\sigma^2_{B,,\max})^2$	$f_{B,\min}$	$(\sigma^2_{B,\min})^2$	$B_{\max}$
Fit [1]	0-5	0	4072.6	0.048020	0.8661	0.04802	>1	$> (\sigma^2_{B,\mathrm{Hi}})^2$	-
Std.Dev.			3223.7	0.003926					
Fit [2]	0-5	0	0	0.050397	0.9361	0.05040	0.9268	0.05008	0.00030443
Std.Dev.				0.003043					
Fit [3]	1-5	0	0	0.045108	0.7778	0.04511	0.7680	0.04479	0.00030399
Std.Dev.				0.012843					

Table S2. Initial parameter values for the stochastic model. The names of the molecular species and rates follow the notation previously used[4]. On the left are the initial concentrations and rate constants for the deterministic model specified in Eq. 3 with no communication between cells in model units. The values are published and released on sourceforge.net[7] under the keyword, vtens_EI_clock1. These parameters have been

converted to units involving molecular counts for the stochastic model. The right column includes the rates and initial species counts for the stochastic model used to initialize MCMC runs.

Molecular species	Deterministic Model Concentrations in model units	Molecular Counts and rates in units of molecular counts/cell
u_1	5.10E-03	1
u_r0	3.99924	112
u_r1	0.442441	13
u_p	4.24E-07	363
v_p	0.0340071	6
f_0	0.356365	1
f_1	0.0824576	0
f_r	4.90E-07	347
f_p	3.0804	512
w	9.24126	91
g_0	6.62E-03	0
g_1	2.59E-06	1
g_r	1.17E-06	260
g_p	1.37E-05	813
Reaction Rates		
A	6.58E-04	6.06E-13
A_bar	0.546986	0.546986
v_1=S_1*u_1	6.16E-02	83.70772
S_3	1.47E-03	3.56911
S_4	2.2396	5453.449
D_1	0.723678	0.723678
D_3	0.299703	0.299703
C_1	0.0428595	4.81E-05
L_1	31.7758	4.244678
L_3	3.02387	0.4850873
D_4	3.23E-03	3.23E-03
D_6	0.15183	0.15183
D_7	0.138387	0.138387
D_8	2.49E-03	2.49E-03
E-2=C_2*v_p	0.1626872	0.1626872
P	19.5648	3.12E-11
Ac	8.49679	1.63E-08
Bc	2.52197	2.52E+00
Sc	1.01E-06	6.67E+01
Lc	1.15E-08	2.23E+00
Dcr	0.219758	2.20E-01
Dcp	0.696903	6.97E-01

Figures S1, S2 , S3, S4, S5, S6, S7, S8, and S9

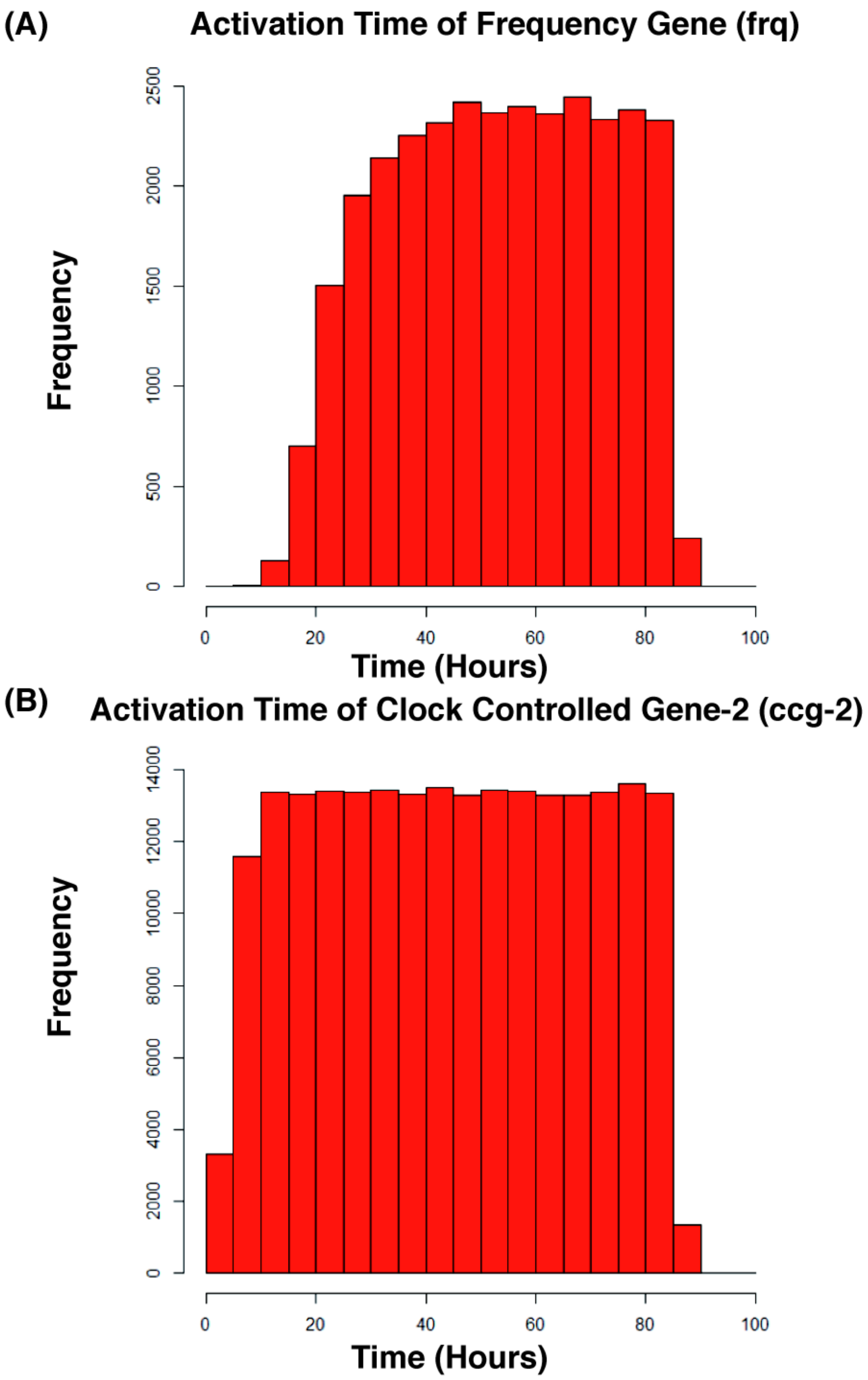

Fig. S1.

Histograms of the times of activation are measured in a window from 30 hours to 115 hours for 1,024 Gillespie trajectories under the best fitting stochastic model, χ^2 = 130.231. The times of activation of these genes are the times of the reaction *frq*^{0} → *frq*^{1} or *ccg-2*^{0} -> *ccg-2*^{1} - 30 hours in each of K=1,024 realizations of the Gillespie stochastic trajectories generated by the model with minimal $\chi^2(\Theta)$ = 130.231 using the Gillespie Algorithm described in Eqns (S1)-(S3). The histogram counts of these two reactions are of all occurrences of *frq*^{0} → *frq*^{1} (Panel A) and of *ccg-2*^{0} -> *ccg-2*^{1} (Panel B). All times of activation are measured from the 30 hour starting time in all 1,024 Gillespie Trajectories. The times of the reactions are only recorded on the time interval from 30 hours to 115 hours so that the time of gene activation can vary from 0 to 85 hours. The zero point in the graph corresponds to 30 hours, and maximum time of activation recorded corresponds to 115 hours.

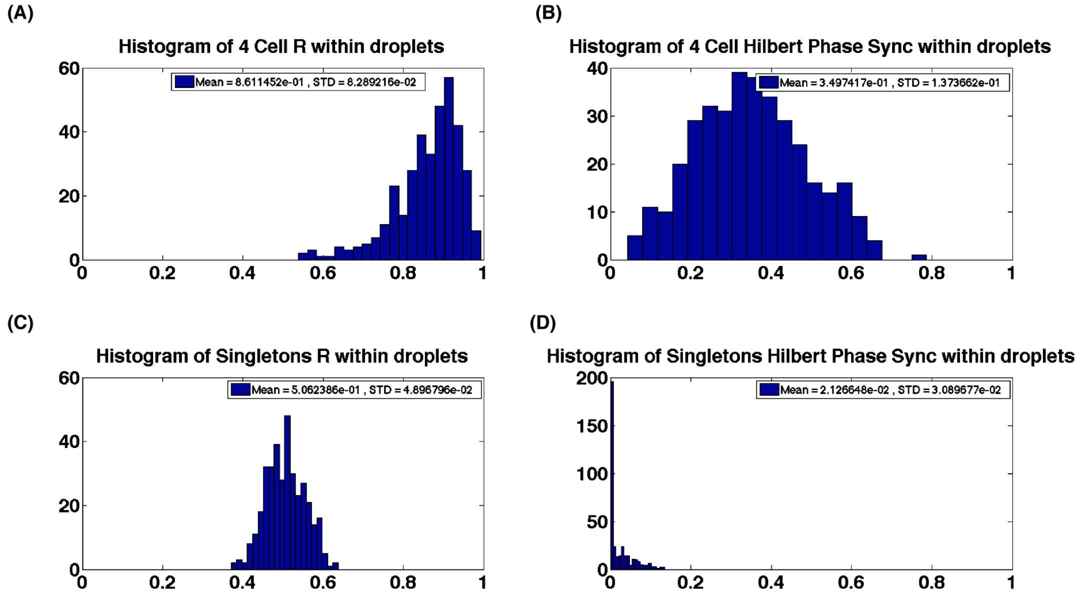

Fig. S2

The synchronization as measured by the "order parameter" R in Garcia-Ojalvo[10] or Hilbert transform phase synchronization[11] are high along the ridge formed at 4 cells per droplet in the Synchronization Surface, shown in Fig. 4C, and they are low when neighbors are replaced with strangers. The figures on the top row, (A) and (B), are from the 4-cell droplet data subset of the main experiment involving 7,903 cells, as shown in Fig. 4C. The bottom row, (C) and (D), is for the control experiment in which neighbors are replaced with strangers.

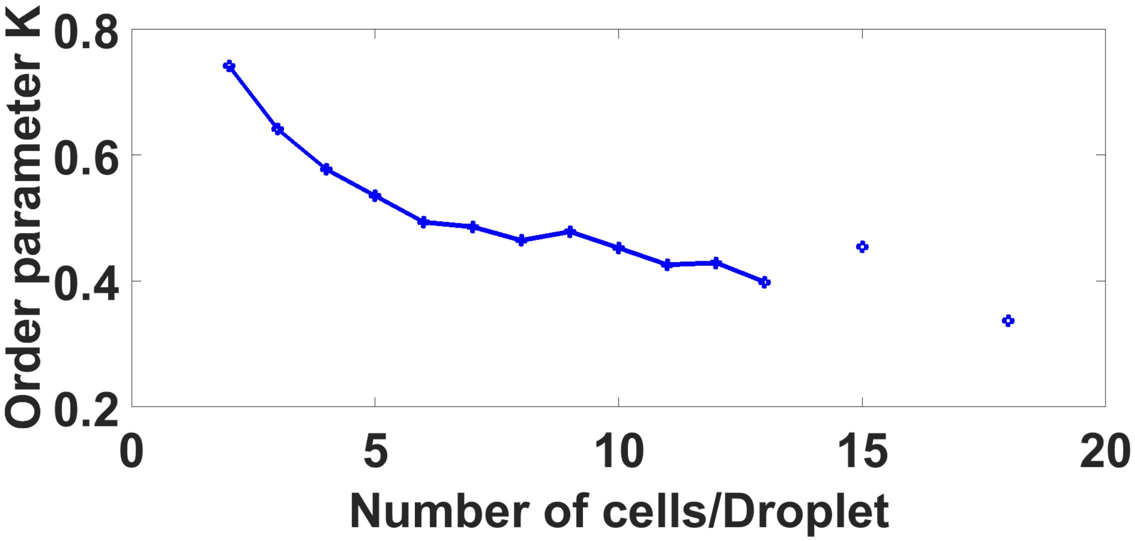

Fig. S3. The Kuramoto order parameter K displays an alternating structure with number of cells per droplet and substantial coherence between oscillators. The order parameter K is the mean absolute deviation of oscillators phases[12]. The last 30 hours of a ten day microfluidics experiment were discarded, and all droplets with less that 80% successful calls on tracking over time were not used. The phase was computed about zero by removing the mean, extracting the discrete Hilbert Phase, and continuizing the discrete Hilbert phase as described in the legend of Fig. 3.

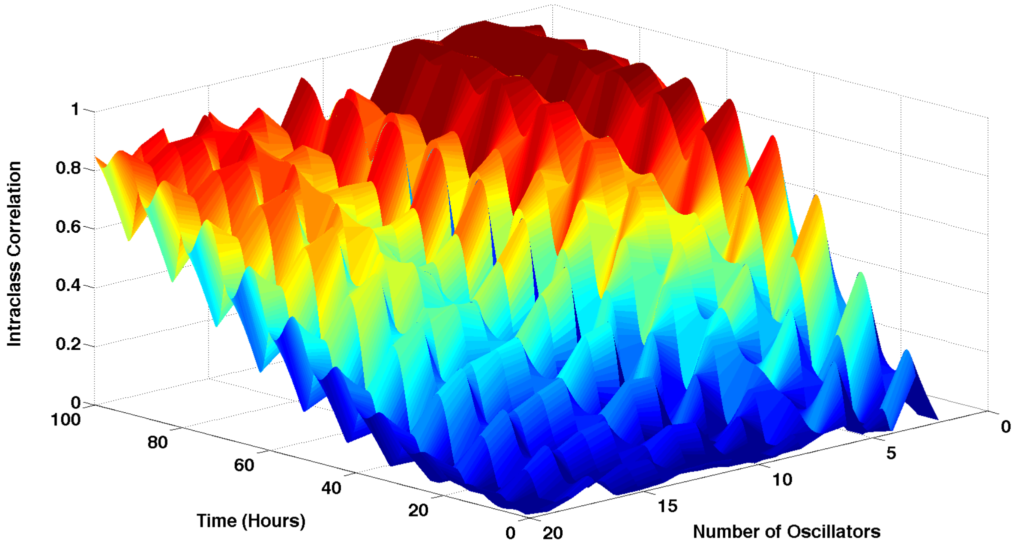

Fig. S4.

The synchronization surface for the Kuramoto phase-locking model[13] as a function of time and the number of oscillators[14]. The model is defined by the equations:

For $s \in [1, N]$,

$$\dot{\varphi}_s = \omega_s + \frac{K}{N} * \sum_{r=1}^{N} \sin(\varphi_r - \varphi_s)$$

The parameters are $\omega_s = \frac{2*\pi}{20}$, K = 0.05.

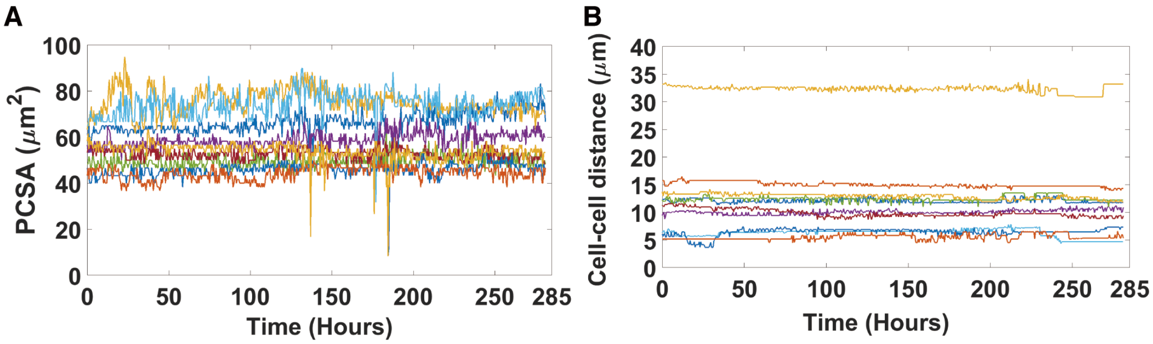

Fig. S5. No change in Projected Cell Surface Area (PCSA) indicates no germination, and no change in cell-to-cell distance indicates no cell fusion. (A) For 10 randomly chosen cells PCSA is followed over 285 h. (B) For 10 randomly chosen cells cell-to-cell distance is tracked over 285 h. Each of these 10 cells and 10 cell pairs were drawn randomly from a larger random sample of ~200 cells or cell pairs that were examined.

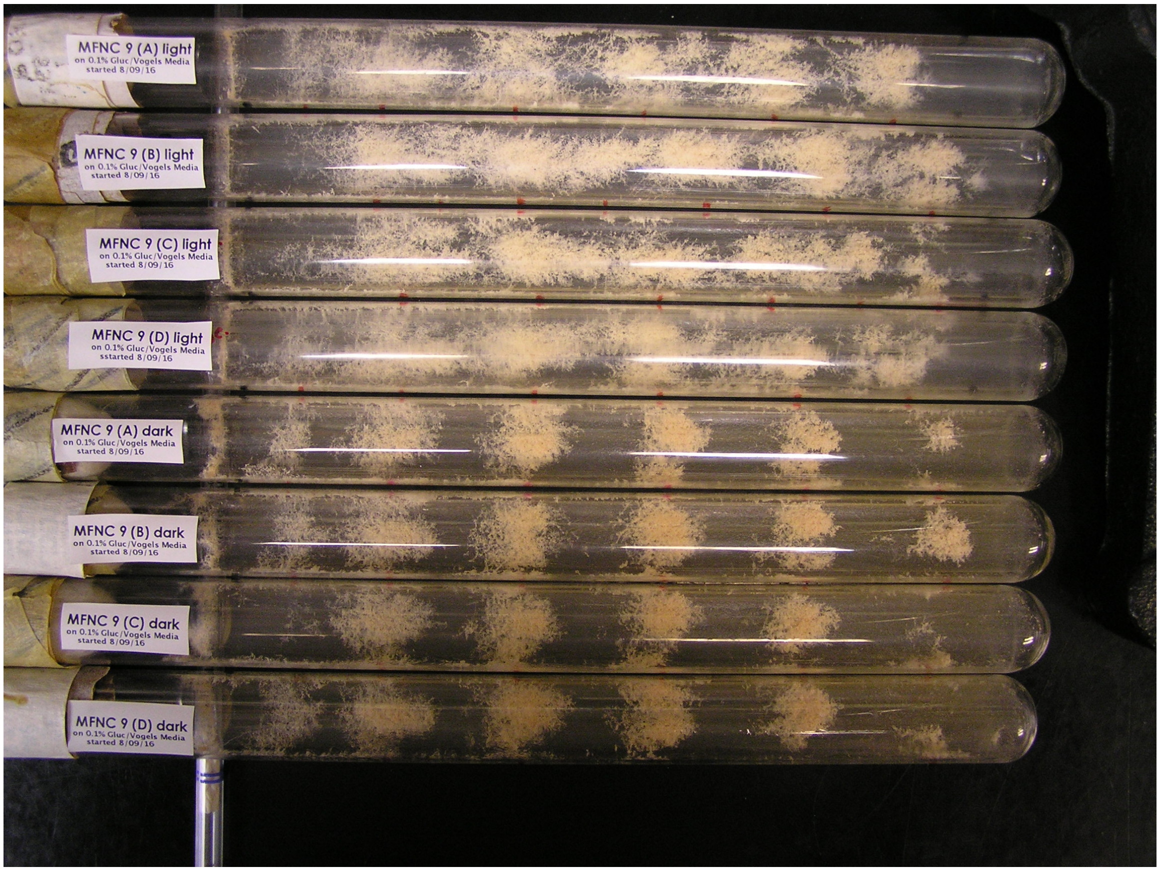

Fig. S6. Race tubes with and without 30 m excitation by light have the same phase and period, but the bands experiencing the 30 m excitation are more irregular. The top four race tubes on top experienced a 30 m excitation from an iPhone. The four tubes on the bottom were in the dark. All tubes were inoculated with the reference strain MFNC9. The exposure time to the iPhone is about 0.5 s.

.

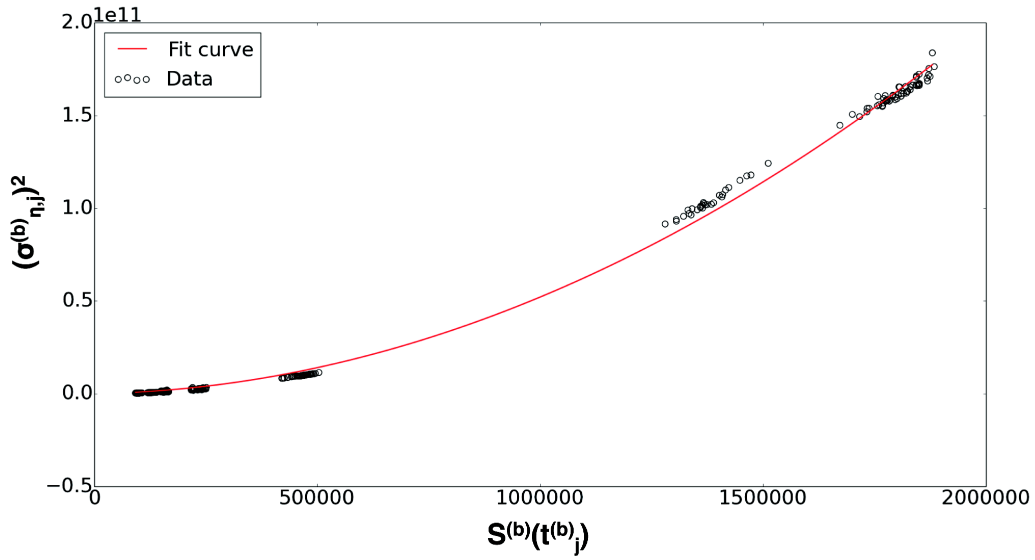

Fig. S7.

The bead signal variance $(\sigma^{(b)}_{\eta,j})^2$ varies quadratically with the mean bead fluorescence signal, $S^{(b)}(t_,^{(b)}{}_j)$ in the six-experiment bead data set, comprising Experiments 0-5. $S^{(b)}(t_,^{(b)}{}_j)$ and $(\sigma^{(b)}_{\eta,j})^2$ were estimated by Eqs. (S21) and (S22), respectively. The fitted red curve is the best fit of Eq. (S19) to the data, subject to the positivity constraints $A^{(b)} \geq 0$, $B^{(b)} \geq 0$ and $C^{(b)} \geq 0$. The best fit parameter values, $A^{(b)}$, $B^{(b)}$ and $C^{(b)}$, are recorded in Table S1. The best fit parameter values, $A^{(b)}$, $B^{(b)}$ and $C^{(b)}$, are recorded in Table S1 as Fit [1].

.

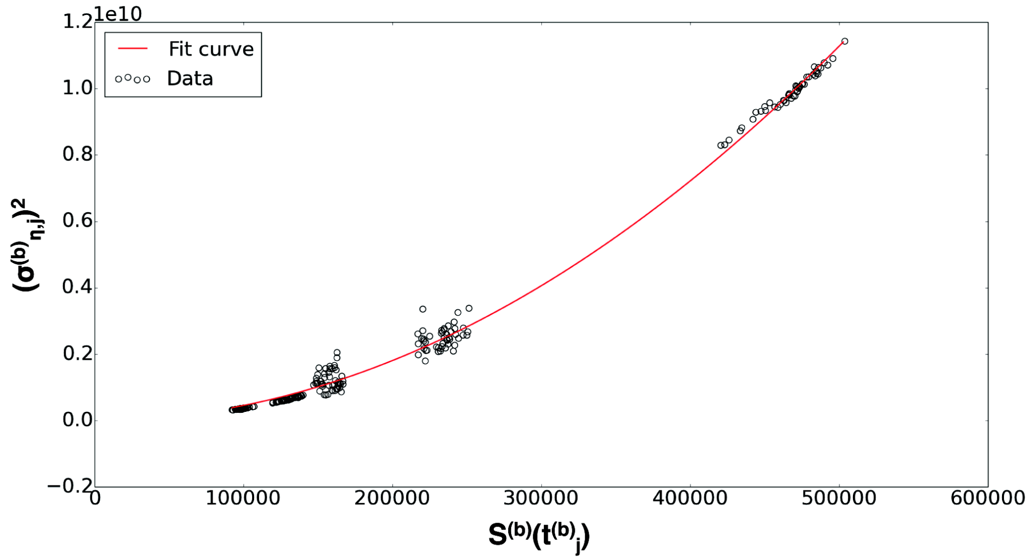

Fig. S8. The bead signal variance $(\sigma^{(b)}{}_{\eta,j})^2$ varies quadratically with the mean bead fluorescence signal, $S^{(b)}(t_{,}^{(b)}{}_j)$ in the five-experiment bead data set, comprising Experiments 1-5. $S^{(b)}(t_{,}^{(b)}{}_j)$ and $(\sigma^{(b)}{}_{\eta,j})^2$ were estimated by Eqs. (S21) and (S22), respectively. The fitted red curve is the best fit of Eq. (S19) to the data, subject to the positivity constraints $A^{(b)} \geq 0$, $B^{(b)} \geq 0$ and $C^{(b)} \geq 0$. The best fit parameter values, $A^{(b)}$, $B^{(b)}$ and $C^{(b)}$, are recorded in Table S1 as Fit [3].

.

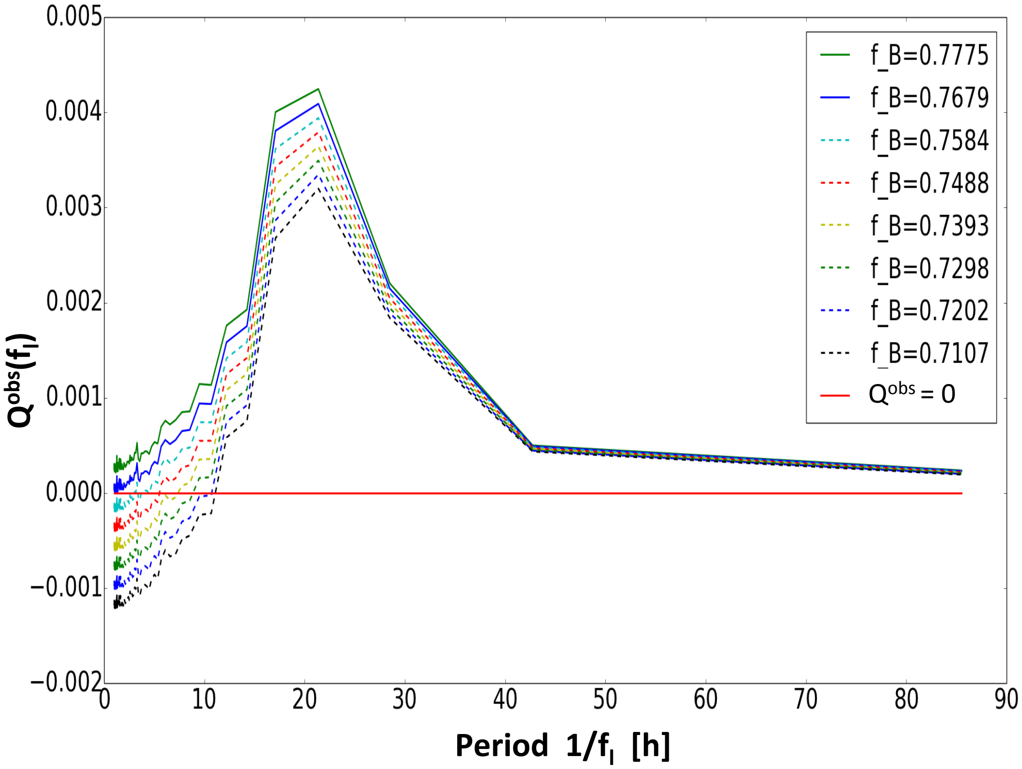

Fig. S9. The observed periodograms, after subtraction of bias due to detector noise, only remain nonnegative (≥ horizontal red line) for a narrow allowed range of the volume fluorescence fraction: $f_{B,min} \leq f_B \leq f_{B,max}$, where $f_{B,min} \cong 0.7680$ and $f_{B,max} \cong 0.7778$, see Table S1, Fit [3]. Each curve is a periodogram, $Q^{\text{obs}}(f_\ell)$, corrected for bias due to detector noise according to Eqs. (S53)-(S61), assuming different values of bead volume fluorescence fraction, f_B and the noise calibration coefficients $A^{(b)}$, $B^{(b)}$ and $C^{(b)}$ recorded in Table S1 as Fit [3].

Caption Video S1

Stability of cell position over more than 213 h in the droplet microfluidics device.

References

1 Lindgren, K. M. Characterization of *ccg-1*, a clock-controlled gene of *Neurospora crassa*. *PhD dissertation. Dartmouth College* (1994).

2 Xia, Y. & Whitesides, G. M. Soft lithography. *Annual Review of Materials Science* **28**, 153-184 (1998).

3 Hartigan, J. A. & Hartigan, P. The dip test of unimodality. *The Annals of Statistics*, 70-84 (1985).

4 Yu, Y. *et al.* A genetic network for the clock of *Neurospora crassa*. *Proc Natl Acad Sci USA* **104**, 2809-2814 (2007).

5 Gonze, D., Halloy, J. & Goldbeter, A. Robustness of circadian rhythms with respect to molecular noise. *Proc Natl Acad Sci USA* **99**, 673-678 (2002).

6 Gillespie, D. T. Exact Stochastic Simulation of Coupled Chemical-Reactions. *J Phys Chem-Us* **81**, 2340-2361 (1977).

7 Al-Omari, A. *et al.* Discovering regulatory network topologies using ensemble methods on GPGPUs with special reference to the biological clock of *Neurospora crassa*. *Access, IEEE* **3**, 27-42 (2015).

8 Izumo, M., Sato, T. R., Straume, M. & Johnson, C. H. Quantitative Analyses of Circadian Gene Expression in Mammalian Cell Cultures. *PLoS Comput Biol* **2**, e136, doi:10.1371/journal.pcbi.0020136 (2006).

9 Battogtokh, D., Asch, D. K., Case, M. E., Arnold, J. & Schuttler, H. B. An ensemble method for identifying regulatory circuits with special reference to the *qa* gene cluster of *Neurospora crassa*. *Proceedings of the National Academy of Sciences of the United States of America* **99**, 16904-16909 (2002).

10 Garcia-Ojalvo, J., Elowitz, M. B. & Strogatz, S. H. Modeling a synthetic multicellular clock: repressilators coupled by quorum sensing. *Proc Natl Acad Sci USA* **101**, 10955-10960 (2004).

11 Kreuz, T. *et al.* Measuring synchronization in coupled model systems: A comparison of different approaches. *Physica D: Nonlinear Phenomena* **225**, 29-42 (2007).

12 Taylor, A. F., Tinsley, M. R., Wang, F., Huang, Z. & Showalter, K. Dynamical quorum sensing and synchronization in large populations of chemical oscillators. *Science* **323**, 614-617 (2009).

13 Kuramoto, Y. *Chemical Oscillations, Waves, and Turbulence*. Vol. 19 (Springer Science & Business Media, 2012).

14 Deng, Z., Arsenault, S., Mao, L. & Arnold, J. Measuring synchronization of stochastic oscillators in biology. *J. of Physics Conference Series, 29th Annual Workshop, 2016, Recent Developments in Computer Simulational Studies in Condensed Matter Physics, Athens, GA, 22-26 February, 2016* **750**, 012001, doi:doi:10.1088/1742-6596/750/1/012001 (2016).
